# Durable and programmable ultrafast nanophotonic matrix of spectral pixels

**DOI:** 10.1038/s41565-024-01756-5

**Published:** 2024-08-12

**Authors:** Tingbiao Guo, Zhi Zhang, Zijian Lin, Jiahan Tian, Yi Jin, Julian Evans, Yinghe Xu, Sailing He

**Affiliations:** 1 Centre for Optical and Electromagnetic Research, Enze-ZJU Joint Lab for MedEngInfo Collaborative Innovation, College of Optical Science and Engineering, Zhejiang University, Hangzhou, People’s Republic of China; 2 Taizhou Institute of Medicine, Health and New Drug Clinical Research; Taizhou Enze Medical Center (Enze), Taizhou Hospital, Zhejiang University, Taizhou, People’s Republic of China; 3 National Engineering Research Center for Optical Instruments, Zhejiang University, Hangzhou, People’s Republic of China; 4 Shanghai Institute for Advanced Study, Zhejiang University, Shanghai, People’s Republic of China; 5 Department of Electromagnetic Engineering, School of Electrical Engineering, KTH Royal Institute of Technology, Stockholm, Sweden

**Keywords:** Nanocavities, Nanophotonics and plasmonics

## Abstract

Locally addressable nanophotonic devices are essential for modern applications such as light detection, optical imaging, beam steering and displays. Despite recent advances, a versatile solution with a high-speed tuning rate, long-life durability and programmability across multiple pixels remains elusive. Here we introduce a programmable nanophotonic matrix consisting of vanadium dioxide (VO_2_) cavities on pixelated microheaters that meets all these requirements. The indirect Joule heating of these VO_2_ cavities can result in pronounced spectral modulation with colour changes and ensures exceptional endurance even after a million switching cycles. Precise control over the thermal dissipation power through a SiO_2_ layer of an optimized thickness on Si facilitates an ultrafast modulation rate exceeding 70 kHz. We demonstrated a video-rate nanophotonic colour display by electrically addressing a matrix of 12 × 12 pixels. Furthermore, inspired by the unique pixel-level programmability with multiple intermediate states of the spectral pixels, a spatiotemporal modulation concept is introduced for spectrum detection.

## Abstract (web summary)

Electrically addressable VO_2_ elements show large resonance shifts during phase transitions, producing a brilliant colour change at a modulation rate of 70 kHz.

## Main

Nanophotonic pixels are the fundamental building blocks of displays, detectors and wavefront manipulation devices. Cavity-based optical devices can manipulate light intensity^1^, phase^2^, polarization^3^ and angular momentum^4^, making them ideal for imaging^5^, displays^6^, spectroscopy^7^, sensing^8^ and thermal management^9,10^. Even with great progress in multiplex engineering, most current devices still exhibit static features limited by the geometric structure or material selection after fabrication, which greatly limits their opportunities for emerging applications such as light detection and ranging^11,12^ and virtual/augmented reality^13^. An ideal tunable photonic platform should simultaneously provide a large modulation capability, a solid state, fast switching, a long life cycle, high scalability and pixel-level programmability. Liquid crystal tunable devices are one of the most promising platforms for versatile functionalities with continuous phase and intensity modulation^14–16^. However, the modulation rate can rarely exceed 1 kHz and is usually polarization dependent. Tunable devices based on the electro-optic effect^17–20^ or carrier injection^21–23^ can work at ultra-high speeds, but they typically have a small modulation depth. Electrochromic or other redox reactions^24–27^ can dynamically control optical transmittance with large intensity contrast. However, the response time of the electrochromic-based modulator is generally slow and the lifetime is always a concern. A comparison among different approaches for electrically programmable photonic devices is listed in Supplementary Section 1.

Electrically driven phase change materials (PCMs) have stimulated many breakthroughs in the field of tunable nanophotonics^28–35^. Among them, chalcogenide PCMs can provide a large modulation depth in the visible and infrared regimes. As a groundbreaking work, Hosseini et al. used conductive atomic force microscopy to tune the phase change of Ge_2_Sb_2_Te_5_ and achieved the first demonstration of a dynamic structural colour display based on PCMs^28^. The authors further developed this technology into arrays of resistive heaters for pixelated switching^30,36^. Later, a reversible structural colour pixel by electrical switching was also developed based on other PCMs^37^. However, high operating temperatures and a large volume expansion during phase changes imposed rigorous requirements for the driving strategy and structure design, greatly limiting the stability and lifetime. Due to this stringent quenching requirement, the realization of large-scale, high-speed, programmable PCM devices is still difficult. Vanadium dioxide (VO_2_) is another PCM with an insulator-to-metal transition at a temperature of ~68 °C (ref. ^38^). It has been widely explored for optical limiters^39,40^, reconfigurable phase plates^41^, dynamic colour filters^42,43^, absorbers^44^ and adaptive thermal^45,46^ regulators. Zhao et al. proposed a dynamic colour display based on VO_2_ that was triggered by a hotplate via a thermal approach with no pixel addressability^43^. This means the patterns were fixed with limited single-purpose functionality once fabricated^47^. Though microheaters have been employed for phase switching recently, especially in integrated photonics (for example, in two studies^32,48^), a thorough exploration for all the properties of pixel-level programmability, multiple stable intermediate states, reversibility and durability is still missing (Supplementary Table 2). Moreover, the response times of reported devices are mainly below the kilohertz range^49^. Pixel addressability across multiple states is the key challenge for a fully functional and versatile platform for display, optical computing and biomedical sensing/imaging, while response time and endurance are the core concerns for practical applications.

In this paper we introduce and demonstrate a general programmable matrix of spectral pixels that possess ultrafast speed, single-pixel addressability with multiple states and a long lifetime. It is composed of VO_2_-based cavities triggered by electrically addressed microheaters and can achieve large spectral/colour modulation through electrothermal heating. By adopting balanced thermal management through optimizing the thickness of the SiO_2_ layer on Si for balanced heating and cooling rates, an ultrafast modulation bandwidth beyond 70 kHz is achieved, nearly two orders of magnitude faster than that of reported devices^50^. Compared to the conventional approach of direct current flow through PCMs, the indirect heating method used here shows better stability even after millions of switching cycles. As a proof of concept of its multidisciplinary applicability, a video-rate nanophotonic display is presented, as well as a spatiotemporal modulation scheme for spectrum detection. With spatiotemporal modulation, the spectrum of the incident light is multiplexed in space across the matrix and in time by multiple intermediate states through the tunable spectral pixels, tackling the trade-off between the footprint and the detection performance in conventional spectrometric devices.

## Design and characterization

**Fig. 1:**
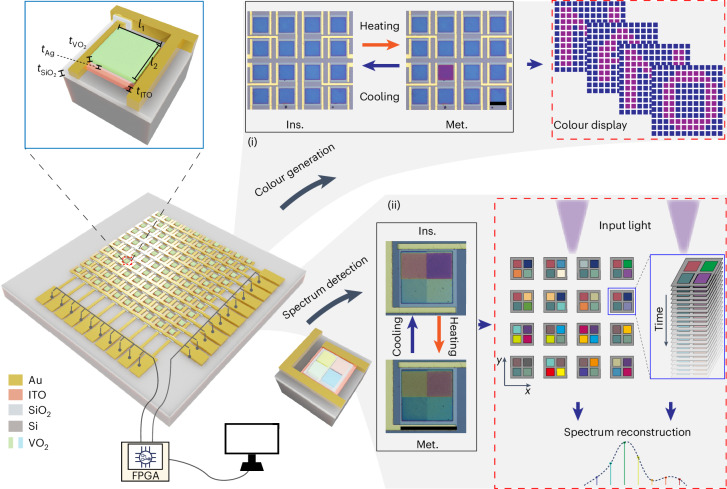
The schematic diagram for the nanophotonic pixel matrix, controlled with an FPGA and computer. Each matrix pixel has a cavity consisting of a metal reflective layer and a VO_2_ layer laid on top of the microheaters made of ITO. The top-left inset shows an enlarged view of a single VO_2_ pixel, with side lengths of *l*_1_ and *l*_2_, VO_2_ thickness of $t_{{\mathrm{VO}}_2}$, ITO thickness of *t*_ITO_, Ag thickness of *t*_Ag_ and SiO_2_ thickness of $t_{{\mathrm{SiO}}_2}$. For colour generation of the nanophotonic matrix (i), the electrically programmable matrix is controlled through the row–column scheme, and the entire device works in the refresh mode for display. For spectrum detection of the nanophotonic matrix (ii), each unit spectral pixel consists of 2 × 2 VO_2_ cavities integrated onto a single heater, which is controlled individually. Every pixel can be regulated to any intermediate state at any time. The black solid boxes in the figure show the optical microscope images of the pixels, and the red dashed boxes show illustrations for display and spectrum detection. Ins., the insulating state; Met., the metallic state. Scale bars, 100 µm in (i) and (ii).

Figure 1 shows the schematic of the proposed spectral pixel matrix. For each unit, a lossy cavity consisting of a metal reflective layer and a lossy VO_2_ layer is adopted for spectral manipulation^1^. A microheater made of indium tin oxide (ITO) is laid beneath each cavity for indirect electrothermal heating. The current will not pass through the VO_2_ directly, and the heat generated by the ITO microheater is transferred through the SiO_2_ layer and then activates the phase transition of VO_2_ in a more uniform way. For a nanophotonic display, the electrically programmable matrix consists of 12 × 12 pixels, where each pixel is controlled through the row–column scheme with a field programmable gate array (FPGA), and the entire device works in refresh mode. For spatiotemporal spectrum modulation, 2 × 2 VO_2_ cavities are integrated onto a single heater, and each heater as a unit spectral pixel is controlled individually by a pair of electrodes. Every pixel could be regulated to any intermediate state at any time. The optical microscope images of the pixels are shown in Fig. 1.

**Fig. 2:**
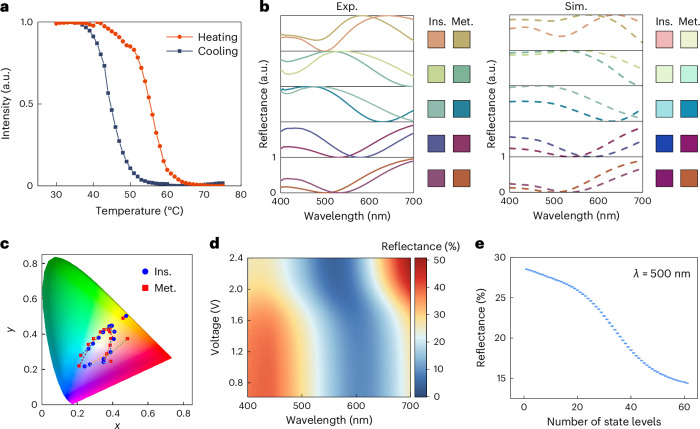
The phase change performance of the VO_2_ cavities. **a**, Heating and cooling curves of the as-prepared VO_2_ material. a.u., arbitrary unit. **b**, Experimental (Exp.) and simulated (Sim.) results of the spectra and colour performance for cavities with various thicknesses of the VO_2_ layer, before and after the phase transition (${t}_{{{\rm{VO}}}_{2}}$ = 30, 40, 60, 80 and 120 nm from bottom to top). The *y* axis for each box has a separate scale of 0 to 1. For simplicity, we only labelled 0 to 1 in the bottom box. **c**, The colour modulation for VO_2_ cavities with different VO_2_ thicknesses in the CIE 1931 colour diagram (the thickness ranges from 10 to 150 nm). Blue discs are for cavities in the insulating state and red squares are for cavities in the metallic state. **d**, The reflective spectra of a VO_2_ cavity (${t}_{{{\rm{VO}}}_{2}}$ = 50 nm) at various intermediate states by applying different input signals. **e**, The measured reflectance for the cavity in **d** at 500 nm at each intermediate-state level over 11 voltage cycles (the error bars indicate the standard deviation of the cycles, and the solid discs indicate the average value).

The as-prepared VO_2_ material was first characterized; Fig. 2a and Supplementary Section 2 show the results. The VO_2_ shows a typical hysteresis effect with a low operating temperature in our device. Various lossy cavities with different thicknesses of the VO_2_ layer were then fabricated. Figure 2b shows the captured colours and reflection spectra for different cavities. As expected, distinct colour differences can be achieved for these structures between metallic and insulating phases. Here, the colour difference (Δ*E*) for each structure before and after the phase transition is calculated to evaluate the quality of colour modulation. The Δ*E* value can be larger than 45 in CIELAB colour space for cavities with a thickness around 40 nm (Supplementary Section 3). The simulated colours and spectra as shown in Fig. 2b agree well with the experimental results. The colour change for more samples is labelled in the International Commission on Illumination (CIE) 1931 colour diagram as shown in Fig. 2c. By varying the thickness of the VO_2_ layer, a full gamut accounting for 40% of standard red–green–blue (sRGB) colour space could be obtained. The calculation and appearance change for different configurations can be found in Supplementary Section 4. Figure 2d shows the heatmap of the reflection spectra when applying various voltages on a single spectral pixel with a VO_2_ thickness of 50 nm. The spectra of all intermediate states have been measured multiple times, and the device can achieve stable intermediate-state colours and spectra under the same temperatures or voltages (Supplementary Section 5 and Fig. 2e, from which we see over 60 intermediate-state levels at a wavelength where the reflectance variation range is large). The device can also work as an intensity or spectrum modulator, and the modulation ratios for different structures can be found in Supplementary Section 6.

## An ultrafast and durable VO_2_ pixel

**Fig. 3:**
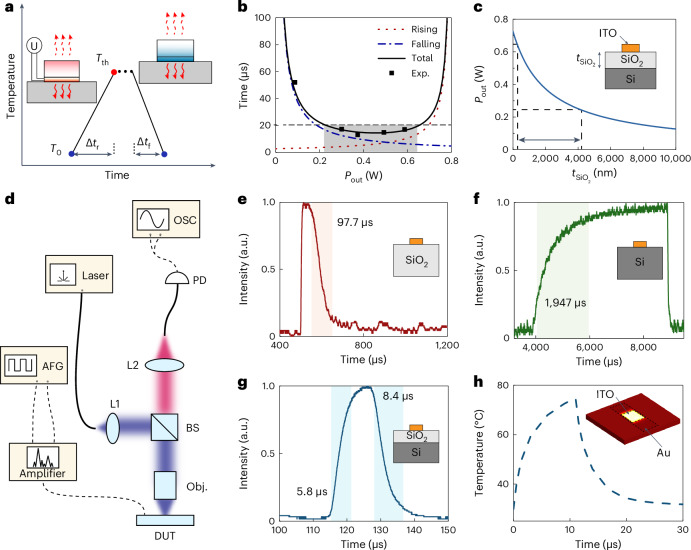
The optimization for the electrothermal response speed of the VO_2_ cavities. **a**, Illustration of the heating and cooling processes. Here *T*_0_ and *T*_th_ are the initial and threshold temperatures, Δ*t*_r_ denotes the rising (heating) time and Δ*t*_f_ denotes the falling (cooling) time. The total time is the sum of the rising and falling times. Here the symbol U represents the voltage source. **b**, The theoretical estimation of rising and falling times for the device as a function of the thermal dissipation power, *P*_out_. The shaded area indicates the *P*_out_ needed for a fast response time below 20 µs, and the square dots are the total time of the experimental results. **c**, The effective output power estimated from the effective thermal conductivity. The arrow indicates the SiO_2_ thickness region required to obtain a thermal dissipation power *P*_out_ within the shaded region in **b**. The inset shows the cross section of an ITO heater on a SiO_2_/Si substrate. **d**, The measurement set-up for the dynamic response. L1(2), lens 1(2); BS, beam splitter; Obj., objective; DUT, device under test; PD, photodetector; OSC, oscilloscope; AFG, arbitrary function generator. **e**–**g**, The measured rising and falling times for devices fabricated on different substrates, namely, SiO_2_ (**e**), Si (**f**) and SiO_2_/Si (**g**). The shaded area is labelled with the time to reach 90% of maximum or minimum intensity. The insets show the cross sections of the heaters on different substrate configurations. **h**, The simulated rising and falling times for the device on a SiO_2_/Si substrate. The inset shows the simulation model. The bright yellow area indicates the hot spot of the temperature.

An important aspect of a dynamic device is the modulation bandwidth, especially for, for example, free-space communication, endoscopic bio-imaging and beam steering applications. In electronic devices, the switching speed of VO_2_ microstructures can reach picoseconds^51^, which is attractive for tunable optical devices. However, in photonic devices, hindered by the large volume of the devices, even a tens-of-kilohertz rate is hard to reach. To better explore the factors for switching speed, we consider both heating and cooling times. As shown in Fig. 3a, two simple equations can be adopted to estimate the heating and cooling processes:

1 $${I}^{2}\times R\times \Delta {t}_{{\mathrm{r}}}=c\times m\times \left({T}_{{{\mathrm{th}}}}-{T}_{0}\right)+V\times H+{P}_{{{\mathrm{rout}}}}\times \Delta {t}_{{\mathrm{r}}}$$

2 $$c\times m\times \left({T}_{0}-{T}_{{{\mathrm{th}}}}\right)-V\times H+{P}_{{{\mathrm{fout}}}}\times \Delta {t}_{{\mathrm{f}}}=0$$

where *I* is the current in the microheater; *R* is the resistance of the microheater; Δ*t*_r_ and Δ*t*_f_ are the rising and falling times for the heating and cooling periods, respectively; *c* is the thermal capacity of the device; *m* is the mass; *T*_0_ and *T*_th_ are the initi al and threshold temperatures, respectively; *V* is the volume of VO_2_; *H* is the latent heat of the phase transition of VO_2_; and *P*_rout_ and *P*_fout_ are the effective thermal dissipation power for the rising (heating) and falling (cooling) periods, respectively. In our simulation *V* × *H* is negligibly small as the volume *V* is small. Here, for simplicity, we assume the effective output power is a constant for both the heating and cooling processes. For a general thermal system, the switching speed can be determined by the thermal capacity *c* and thermal conductance *g* as *τ* = *c*/*g*. This implies that the larger the thermal conductance, the faster the response is. However, in our case, the thermal source is limited by a voltage supplier. To ensure the device can be heated over the temperature required for the phase transition of VO_2_, the thermal conductance needs to be in a reasonable range.

Considering all the aspects listed above, a reasonable effective output power is critical for balanced and fast rising and falling times (Fig. 3b). By controlling the effective output thermal power within a range from ~0.2 W to 0.6 W, the response time of this device can be squeezed to less than 20 µs, much faster than previous VO_2_-based reconfigurable devices. Considering that thermal conduction is the main heat dissipation channel in our device (Supplementary Section 7), we manipulate the effective output power by inserting a silicon oxide layer between the device and the silicon substrate and varying its thickness for a balanced heat dissipation rate. Figure 3c shows the calculated relationship between the effective output thermal power, *P*_out_, and the thickness of SiO_2_, ${t}_{{{\rm{SiO}}}_{2}}$. By adopting a SiO_2_ layer with a thickness ranging from hundreds of nanometres to several micrometres on a silicon substrate, our device could balance the heating and cooling processes and possess an ultrafast response. Calculations are in Supplementary Section 8.

Figure 3d shows the experimental set-up for the response time characterization, which mainly consists of a light source, a signal generator with a voltage amplifier and an optical detector with an oscillator. The measured responses for three different substrates are shown in Fig. 3e–g. A thick glass substrate is beneficial for a fast rising time but leads to a slow falling time (Fig. 3e). The rising and falling times are 6.6 µs and 97.7 µs, respectively. A pure silicon substrate (Fig. 3f) can provide a fast heat dissipation rate and hence a fast falling time, but this comes along with a long rising time. With a solely silicon substrate, the switching times are 1,947 µs and 85 µs for the rising and falling periods, respectively. Neither case can give a fast response for both the falling and rising periods simultaneously. By using the silicon substrate with a 2-µm-thick SiO_2_ cap layer, one can manipulate and balance the heating and cooling processes, giving fast rising and falling times of 5.8 µs and 8.4 µs, respectively, which are 335 and 11 times faster than those in the silicon/glass conditions as shown in Fig. 3g. Experimental results for other SiO_2_/Si thickness configurations are also labelled in Fig. 3b, fitting well with the theoretical results. We conducted an electrothermal simulation (Fig. 3h) based on the optimal configuration, and the response times for the heating and cooling processes are consistent with the experimental results. By further shrinking the pixel size, our device has the potential to operate in regimes of a few megahertz and with a decreased driving voltage. Supplementary Section 9 provides more detailed information about the simulation and experiment.

**Fig. 4:**
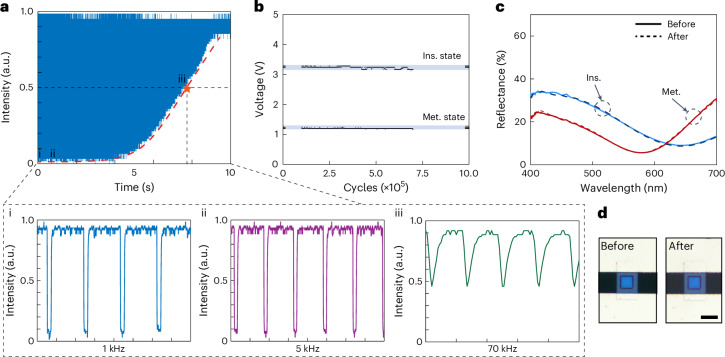
The dynamic response and the life cycle of the VO_2_ cavities. **a**, Dynamic response as a function of varying modulation frequency. Panels i–iii are zoomed-in views of the areas labelled in the main panel and show the responses for 1 kHz, 5 kHz and 70 kHz modulated input signals, respectively. **b**, The durability of our device for one million cycling times. The device is irradiated with a 532 nm laser while an on–off electrical signal is applied to an ITO microheater. A photodetector is used to monitor the reflected light. The vertical label shows the voltage output at voltages around 3 V and 1 V (of an oscilloscope connected to the photodetector) when the electrical signal is on and off, respectively. The shaded areas indicate the variations of the outputs. **c**, The reflectance before and after one million times of switching. **d**, The appearance before and after one million times of switching. The colour difference Δ*E* is less than 0.65 before and after one million times of switching. Scale bar, 20 µm.

To further validate the frequency response of the device, we carried out modulation measurements on the sample by the logarithmic increase of voltage-biased switching frequency, from 100 Hz to 100 kHz, with a total sweep time of 10 s. The measured 3 dB cut-off frequency can be over 70 kHz (Fig. 4a). We also plot the signal responses at frequencies of 1 kHz, 5 kHz and 70 kHz, confirming the ultrafast response modulation. We evaluated the durability of the device by applying a signal with a frequency of 1 kHz and a duty cycle of 10%. After switching one million times, we saw no notable change in the response of our device (Fig. 4b). The robustness of the present switching device was also confirmed by measuring the reflection spectra of the sample before and after the experiment. The small changes in the reflection spectra and appearance (Fig. 4c,d) indicate that the sample did not undergo notable deterioration. This ultra-long lifetime is mainly attributed to the low threshold temperature of VO_2_ devices, alleviating the burden for a microheater to trigger the phase transition. For the present device, the trigger current for the phase transition is around 37.5 mA.

## Structural colour display by row–column addressing

**Fig. 5:**
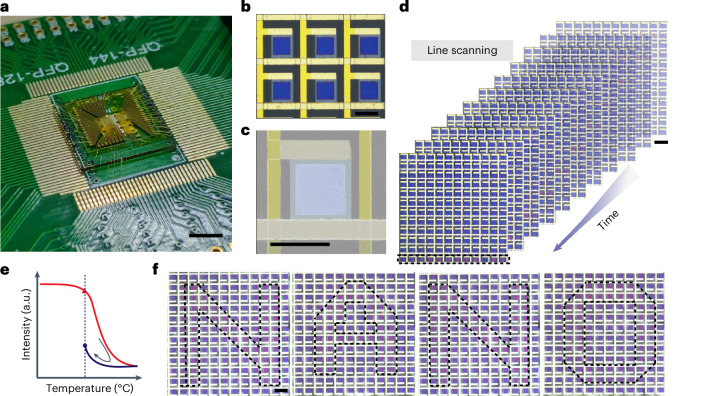
Demonstration of a structural colour display. **a**, Photograph of the prototype of the structural colour matrix. **b**,**c**, The optical images (**b**) and a false-colour scanning electron microscope image (**c**) of the structural colour pixels. **d**, Line-scanning screenshots at different times. **e**, The principle of a non-volatile display. The red triangle marks the starting point, and the blue disc is the final state of the device. **f**, The patterns of sequentially appearing letters (one letter pattern at a time) showing ‘NANO’, based on the hysteresis effect. Dashed lines are included only for ease of observation. Scale bars, 1 cm in **a**, 50 µm in **b** and **c** and 100 µm in **d** and **f**.

We adopted a row–column addressing scheme to achieve programmable control of the 12 × 12 pixels for a prototype structural colour display^52^. More information about the design can be found in Supplementary Section 10. Figure 5a shows the image of our sample bonded to a printed circuit board, and the row and column electrodes are connected to a switch matrix and FPGA. More information about the matrix control can be found in Supplementary Section 11. Figure 5b,c shows the zoomed-in optical microscope and scanning electron microscope images of the chip. The pixels are scanned and displayed line by line with FPGA control. Figure 5d gives a series of line-scanning screenshots with a time interval of 100 ms. More information about single-pixel scanning and line scanning is included in Supplementary Videos 1 and 2. In addition to the high-speed response, based on the hysteresis effect of VO_2_, we also demonstrated a non-volatile display. The whole device was first placed on a hotplate at ~54 °C. Then a 1.1 V pulsed signal was applied to some of the pixels to trigger the phase transition process. Due to the hysteresis effect (Fig. 5e), the pixels with a voltage on underwent a different colour change. Figure 5f shows a pattern of ‘NANO’ created by this effect. The pixel-to-pixel uniformity of the matrix was also characterized as shown in Supplementary Section 12.

## Spatiotemporal spectrum detection

**Fig. 6:**
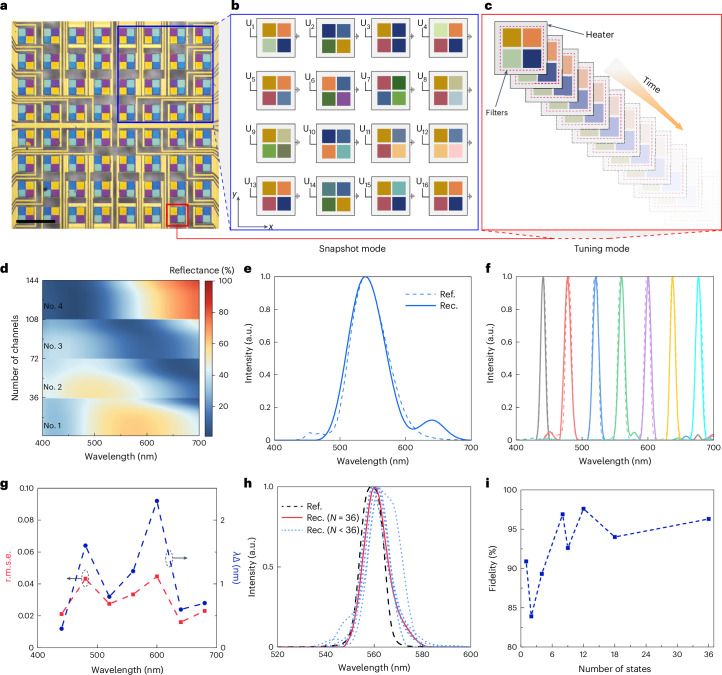
Spectrum detection through spatiotemporal modulation. **a**, The optical image of the tetrachromatic filter matrix for spectrum detection. Scale bar, 200 μm. **b**, An enlarged view of a chosen region (a large part of the filter matrix, indicated by a blue box in **a**), where each unit filter is activated to a specific intermediate state with a different specific voltage, labelled *U*_*n*_. This illustrates the principle for spectrum detection in snapshot mode. **c**, An enlarged view for a single unit filter (indicated by a red box in **a**), which is activated to different intermediate states with different voltages in a time sequence. This illustrates the principle for spectrum detection in tuning mode. **d**, The reflective spectra of the tetrachromatic filter used for spectrum reconstruction. Each filter is set to 36 intermediate states, and the whole channel number (the total number of filters multiplied by the total number of intermediate states) used for spectrum reconstruction is 144. **e**,**f**, The reconstructed results for broadband (**e**) and narrowband (**f**; with a bandwidth about 10 nm) signals. The dashed lines (Ref.) are the ground truth measured by a commercial spectrometer, and the solid lines are reconstructed results (Rec.). **g**, The reconstruction performance for narrowband signals. **h**, The reconstructed performance with different numbers of states. The dashed line is the ground truth measured by a commercial spectrometer; the solid line is the reconstructed spectrum with a total number of states, *N* = 36; and the dotted lines are reconstructed spectra with *N* = 1, 2, 4, 8, 9, 12 and 18. **i**, The corresponding fidelity calculated from **h**. The dashed lines in **g** and **i** only provide connections to the points to show the general tendency.

To show its versatility, the programmable matrix of spectral pixels was then employed for spectrum detection. In conventional miniaturized spectrometer systems, the unknown spectrum is reconstructed either by grating/filter arrays, spatially (for example, as in one study^53^ and the references therein), or by tunable filters temporally (for example, as in another study^54^ and the references therein). These methods present the dilemma of either enlarging the footprint or deteriorating the detection performance (for example, spectral resolution, response time or working wavelength range). By harnessing the multiple intermediate states and the pixel-addressing feature of the matrix, we tackled this trade-off with a unique spatiotemporal modulation scheme. The design approach involves integrating 2 × 2 VO_2_ tunable filters on a single ITO heater to form a unit pixel as shown in Fig. 6a. These tetrachromatic filters act like RGGB Bayer filters (the filter pattern is half green, one quarter red and one quarter blue) but offer reconfigurable responses. Consequently, the scheme possesses spectrum detection aided by advanced reconstruction algorithms (Supplementary Section 13 for the principle of colour and spectrum sensing with these active spectral filters). The pixelated tuning feature combined with multiple states provides versatile strategies for spectrum detection with flexible degrees of freedom over the spatial, spectral and temporal domains. As an example, the strategies of snapshot mode and tuning mode for spectrum detection, as illustrated in Fig. 6b,c, showcase the flexibility and power of this spatiotemporal filter matrix. For snapshot mode detection, sixteen unit pixels with each pixel triggered at a unique intermediate state are employed, enabling a real-time overview of the input spectral content through the reconstruction process. For tuning mode detection, a single unit pixel is cycled through various intermediate states, allowing for a fine-tuned spectral response with an ultracompact footprint.

Figure 6d shows the reflection spectra for four filters in a unit pixel, and Fig. 6e–g shows the spectra reconstructed by these filters for both narrowband and broadband input signals. All reconstruction agrees well with the reference spectra recorded by a commercial spectrometer with a root mean square error (r.m.s.e.) of less than 0.05 (narrowband) and 0.06 (broadband), with the largest peak difference (Δ*λ*) less than 2.5 nm. The consistency between the reconstructed and reference spectra underscores the versatility and reliability of this spectral detection system. Furthermore, we also compared the reconstruction fidelity with different intermediate states (Fig. 6h). Using only four intermediate states, a fidelity of around 90% can be achieved (Fig. 6i), which greatly accelerates the reconstruction process without compromising the quality of the results. More experimental details and the reconstruction process can be found in Supplementary Section 14.

## Conclusions

In conclusion, we have demonstrated a video-rate nanophotonic display with VO_2_-based active pixels and introduced a unique spatiotemporal modulation for spectrum detection. By further optimizing the thermal capacity along with an advanced driving scheme^55^, our device has the potential to operate in megahertz regimes. As the matrix size increases, the increasing number of sneak current paths will lead to substantial waste power dissipation for the structural colour display (Supplementary Section 15). Moreover, the thermal cross-talk between adjacent pixels also requires careful consideration (Supplementary Section 16). Transistors could be incorporated into the pixel to improve the selectivity and suppress the sneak path effect, particularly in high-power-consuming devices. In Supplementary Section 17, we compare our study with previously reported dynamic structural colour based on PCMs. As for spectrum detection, the current prototype uses an 8 × 8 matrix of active spectral pixels, which could be expanded to a larger scale with the more sophisticated read-out circuits widely used in current image sensors. With megapixels, one could achieve an on-demand spectral imaging system, by customizing the region of interest with the hybrid strategy of snapshot mode and tuning mode. With a more complicated design strategy, the spatiotemporal filter could also work in transmissive mode or be extended to infrared bands (Supplementary Section 18). Due to its compatibility with present-day Bayer filters, the spatiotemporal concept is expected to generate new detection architectures for imaging and sensing applications by adapting other active platforms such as two-dimensional (2D) materials^56^, liquid crystals^57^ and semiconductors^58^. This enables many programmable devices that have customized requirements, such as multifocal lenses, biomedical sensing and imaging, high-speed and solid-state optical switches, video displays and light detection and ranging.

## Methods

### Numerical simulation

The optical reflectance of our devices was simulated by Ansys finite-difference time-domain software with a 2D model to reduce the simulation time. In the simulation, the refractive indices of VO_2_ were adopted as the measured values. Other materials were adopted from the software library. Electrical and thermal responses were simulated with COMSOL. The measured conductivity (5 × 10^4^ S m^–1^) of the ITO was used in the simulation. All other material properties were adopted from the built-in library. The reflective colours and colour differences were obtained by homemade MATLAB scripts.

### Design and fabrication

The SiO_2_/Si substrate was fabricated by plasma-enhanced chemical vapour deposition (made by Surface Technology Systems Ltd, model M/PLEX CVD) from a bare silicon substrate. The whole device fabrication process was as follows. First, the ITO heater patterns were fabricated by UV photolithography (Suss, MA6 mask aligner), followed by the sputtering and lift-off process for the ITO heater. After that, we fabricated the Cr/Au electrode/wire patterns with the same method, using an electron-beam evaporator (Denton Vacuum, Explorer). Then the lossy cavities were fabricated by UV photolithography, followed by depositing a thin SiO_2_ layer by plasma-enhanced chemical vapor deposition. Then Cr, Ag and V layers were sputtered sequentially by a magnetron sputtering machine (Kurt J. Lesker, PVD75). A VO_2_ layer was formed by thermal annealing at 400 °C for 30 min in a thermal oxidation furnace (ATV-tech, PEO601). The vanadium, gold, silver and chromium targets with a diameter of 50.8 mm were purchased from ZhongNuo Advanced Material (Beijing) Technology. The ITO target was purchased from JiuYue Advanced Material Technology.

### Measurement and characterization

The optical images were captured by an Olympus BX53M microscope. To measure the reflectance of the samples, a modified Olympus BX53M microscope mounted with a fibre and a spectrometer (Ocean Insight, QE Pro) was used. The electrical signal was generated using a signal generator (SIGLENT, SDG1062X) and a power amplifier (Aigtek, ATA-105). The response time was measured by a photodetector (Thorlabs, PDA100A-EC) connected with an oscilloscope (RIGOL, DS1202). For the durability measurement, a 532 nm laser (Lasever) was used to irradiate the sample, and we measured the reflectance of the sample using an amplified photodetector (Thorlabs, PDA100A-EC) with a gain of 40 by applying an a.c. voltage signal. The output of the photodetector was then fed to an oscilloscope.

## Online content

Any methods, additional references, Nature Portfolio reporting summaries, source data, extended data, supplementary information, acknowledgements, peer review information; details of author contributions and competing interests; and statements of data and code availability are available at 10.1038/s41565-024-01756-5.

## Supplementary information

Supplementary Information Supplementary Figs. 1–18, Tables 1–4 and Discussion.

Supplementary Video 1 The dot-scanning demonstration with the proposed spectral matrix.

Supplementary Video 2 The line-scanning demonstration with the proposed spectral matrix.

## Data Availability

The data that support the findings of this study are available via figshare at 10.6084/m9.figshare.26183465.v2 (ref. ^59^).

## Code availability

The code used to generate the reflective colours and colour differences can be available from the corresponding author upon reasonable request.
