# Supplementary material for: Durable and programmable ultrafast nanophotonic matrix of spectral pixels
Source: Nat Nanotechnol. 2024 Aug 12;19(11):1635–43. doi: 10.1038/s41565-024-01756-5 (PMC11567887; doi:10.1038/s41565-024-01756-5)
Supplement: Supplementary file 1 — Supplementary Figs. 1–18, Tables 1–4 and Discussion. [file 41565_2024_1756_MOESM1_ESM.pdf]

nature nanotechnology

Supplementary information https://doi.org/10.1038/s41565-024-01756-5

Durable and programmable ultrafast nanophotonic matrix of spectral pixels

In the format provided by the authors and unedited

Table of Contents

1. Electrically-programmable approaches for nanophotonic devices 3

1.1 Liquid crystals 3

1.2 Carrier injection 3

1.3 Phase-change materials 3

1.4 Pockels effect 3

1.5 Thermo-optic (TO) effect 3

1.6 Microheater-triggered PCM devices 4

2. The characterization of the VO_2 materials 4

2.1. The refractive index of the VO_2 materials. 4

2.2. The Raman spectrum of the VO_2 materials. 5

2.3. The XRD spectrum of the VO_2 materials. 5

3. The color difference of the lossy cavity with different thicknesses 5

4. The color calculation and appearance change for different cavity configurations 6

5. The repeatability and the generation of intermediate states 6

6. The intensity modulation of the lossy cavity 7

7. The heat dissipation channels for our devices 8

8. The heat transfer model and calculation 8

9. Simulation of the dynamic response 9

10. Layout design of the 12×12 matrix and pixel control 10

11. Implementation of matrix control 11

12. The uniformity of the matrix 11

13. The principle of color and spectrum sensing by the active spectral filters 12

13.1 Color sensing 12

13.2 Spectrum sensing 13

14. The spectrum detection and reconstruction process 14

14.1. Setup for spectrum detection 14

14.2. Calibration of the filter arrays 14

14.3. Measurement 14

14.4. Reconstruction 14

15. The sneak current in the cross-bar scheme 15

16. The simulated and experimental results for thermal crosstalk 20

17. The PCM-based structural color filters 20

18. The transmissive spectral filter and filters for the infrared band 21

1. Electrically-programmable approaches for nanophotonic devices

Here we have listed typical approaches in detail for electrically-programmable nanophotonic devices.

1.1 Liquid crystals

Liquid crystal is a widely used material in tunable metasurfaces. They can adjust their response by changing the refractive index of the surrounding medium or changing the polarization of incident light. In addition, the changes in liquid crystals are usually based on the Fréedericksz transition process, which is relatively slow and limits the switching speed of liquid crystals, usually below 1 kHz. Meanwhile, the sensitivity of liquid crystals to polarization also limits their applications in certain situations.

1.2 Carrier injection

Metasurfaces can change their scattering characteristics by controlling their carrier concentration. The modulation of changing carrier injection concentration often exhibits an ultra-fast response. However, its drawbacks are relatively small modulation depth and typically only working in a reflective state.

1.3 Phase-change materials

Phase change materials (PCM) have been widely introduced in optical devices in recent years to provide tunability. Common phase change materials are mainly divided into two categories, one is sulfur-based phase change materials, and the other is vanadium dioxide materials used in our article. The characteristic of chalcogenide-based phase change materials is that the transition from amorphous to crystalline requires exceeding the crystallization temperature, while the transition from crystalline to amorphous requires high temperatures (>600 °C) and a rigorous quenching process. The high temperature and quenching process make its manufacturing process complex and not conducive to large-scale pixelated preparation. Although the theoretical switching speed of chalcogenide-based phase change materials is very fast (ns), in practical applications, a slower crystallization process is usually required to fully crystallize the device, thereby avoiding material deterioration during the amorphization process.

1.4 Pockels effect

Materials lacking inversion symmetry undergo refractive index changes when an electric field is applied. Pockels effect can modulate the environment around the surface of elements to regulate their response. The main drawback of this tuning method is that it can only be achieved in specific materials lacking inversion symmetry, and this response is usually small.

1.5 Thermo-optic (TO) effect

Thermo optical tunable photonic devices can provide almost zero variation in optical loss while achieving moderate changes in optical index at the cost of relatively high power consumption. The selection of materials with high thermal optical coefficients is limited, and the speed is slow due to the properties of the materials.

A comparison between different metasurface modulation methods can be found in Table S1.

Table S1. Techniques used for electrically tuning nanophotonic devices

Refs.	Method	Working Mode	Modulation Depth	Speed	Pixel	Durability	Number of levels
[1]	Liquid crystals	R/T	Medium	Slow	NO	N. A.	Medium
[2]	Carrier injection	R	Low	Fast	NO	N. A.	Low
[3]	Electro-optic	R	Low	Fast	NO	N. A.	Medium
[4]	Electro-optic	R	Low	Fast	NO	N. A.	Low
[5]	Electrochromic	R	High	Low	NO	N. A.	Low
[6]	TO	R/T	Low	Low	YES	Medium	Low

[7]	VO_2	R	High	Medium	YES	N. A.	Low
[8]	GST	R	Medium	Medium	NO	Low	Low
[9]	GSST	R	Medium	Low	NO	Low	Medium
[10]	VO_2	R	High	Medium	YES	N. A.	Low
[11]	Sb_2Se_3	T	Medium	Medium	YES	Medium	Medium
[12]	GST	R	High	Medium	NO	Low	Low
**Our Work**	**VO_2**	**R**	**Medium**	**Fast (>70 kHz)**	**YES (144)**	**High (>1000,000)**	**High (>60)**

Notes: T: Transmissive; R: Reflective; N. A.: Not applicable

1.6 Microheater-triggered PCM devices

In particular, there are many demonstrations of PCM-based devices using microheaters for phase change. However, they rarely explored pixel-addressability, reversibility and endurance. Especially, pixel-addressability is the key challenge for a fully functional and versatile platform for display, computing and sensing and the response time and endurance are the core concerns for practical applications. Here we have listed the representative works for microheater-triggered PCM devices.

Table S2. The microheater-triggered PCM nanophotonic devices.

**Refs.**	**PCM Type**	**Pixel-addressability**	**Life cyclability**	**Speed**	**Size (μm²)**	**Number of levels**	**Year**
[8]	GST	NO	100	10 kHz	10×0.06	3	2021
[9]	GSST	NO	40	2 Hz	100×100	10	2021
[13]	Sb_2S_3	NO	N. A.	N. A.	100×100	NO	2021
[14]	$Ge_{20}Te_{80}$	NO	100	N. A.	50×50	NO	2021
[15]	VO_2	NO	N. A.	3 Hz	200×100	4	2021
[12]	GST	NO	50	5 kHz	10×10	6	2022
[7]	VO_2	**YES**	N. A.	1.2 kHz	40×10	6	2022
[10]	VO_2	**YES**	N. A.	N. A.	8×5	3	2022
[16]	Sb_2Se_3	**YES**	1100	12 kHz	30×30	10	2023
[17]	GSST	NO	1250	1 Hz	200×240	N. A.	2024
[18]	Sb_2S_3	NO	1600	5 Hz	30×0.45	32	2024
[19]	Sb_2Se_3/ $Ge_2Sb_2Se_4Te_1$	NO	1500	10 Hz	20×10	180	2024
**Our work**	**VO_2**	**YES**	**>1,000,000**	**>70 kHz**	**155×120**	**>60**	**2024**

2. The characterization of the VO_2 materials

2.1. The refractive index of the VO_2 materials.

The refractive index for VO_2 was measured by ellipsometry (Horiba, UVISEL).

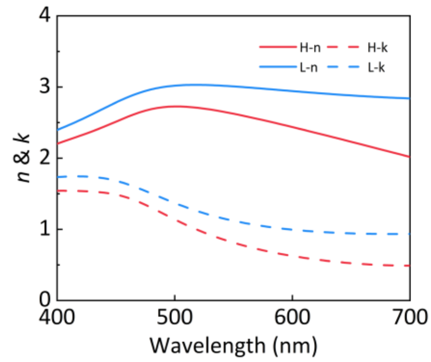

Figure S1. The refractive indices of the VO_2 at low (room temperature) and high (80°C) temperatures. Blue lines for Low temperature and Red lines for High temperature. Solid lines for the real part and dashed lines for the imaginary part.

2.2. The Raman spectrum of the VO_2 materials.

The Raman spectra of our sample are very close to those of vanadium dioxide in many literature.[20,21]

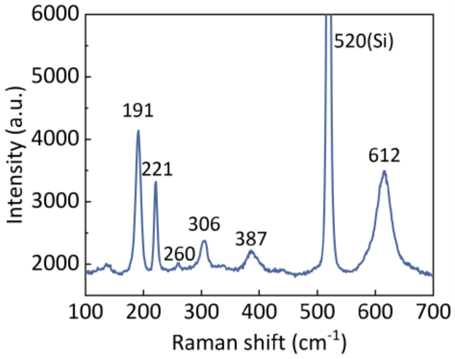

Figure S2. The Raman spectrum of the VO_2 material.

2.3. The XRD spectrum of the VO_2 materials.

XRD spectrum of the Vanadium layer after annealing at 400 degrees, each diffraction peak of VO_2 is attributed to the scattering of the atomic lattice. The XRD of the high-temperature annealed sample is consistent with the typical VO_2 (PDF # 79-1655), and the small deviation of the overall peak may be caused by uneven sample preparation and the need for instrument calibration during sample measurement.

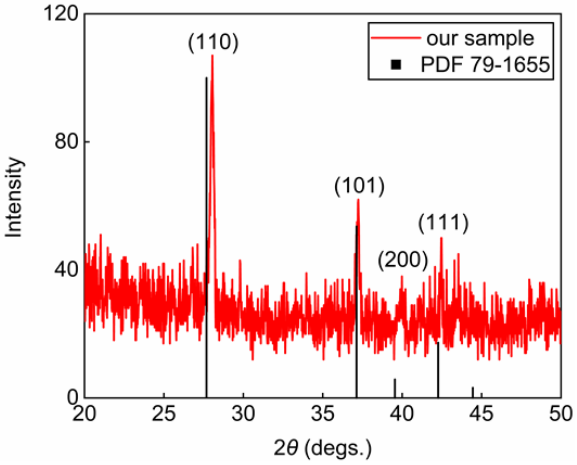

Figure S3. The XRD spectrum of the VO_2 material.

3. The color difference of the lossy cavity with different thicknesses

To better reveal the color differences before and after the phase transition of various cavities, we calculate the color difference ΔE using the formula:

$$\Delta E = \sqrt{\Delta L^{*2} + \Delta a^{*2} + \Delta b^{*2}}$$

The results are shown below. We can observe that the color differences for structures with VO_2 thicknesses of 30, 40, and 140 nm are significant, with a color difference exceeding 40. Based on the light sources and color perception by the human eye, we ultimately chose a structure with a thickness of 40 nm for display. Even in cases where the structure is very small, the changes in blue and rose red can be clearly distinguished by the human eye.

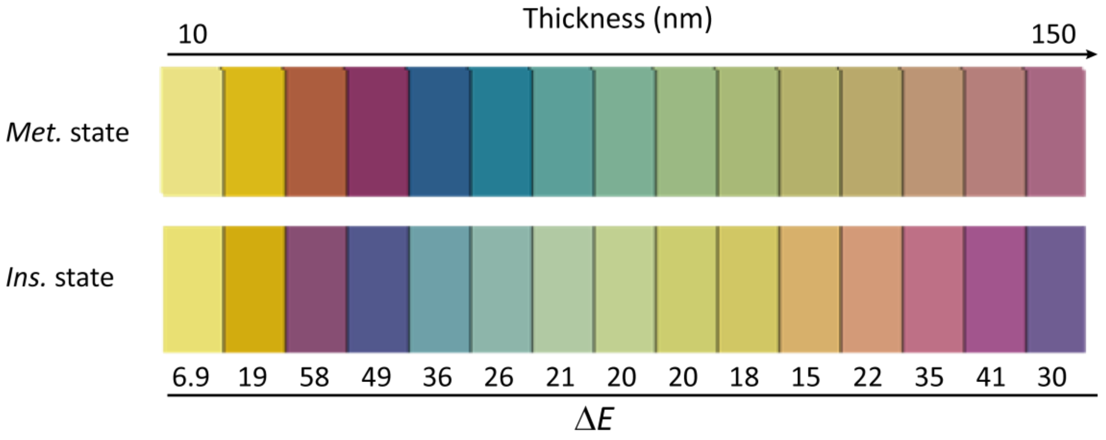

Figure S4. The calculated color difference for various thicknesses of VO_2.

4. The color calculation and appearance change for different cavity configurations

For color reconstruction in Figure 2, we derived the colors from reflection spectra by decomposing them with the CIE standard color matching functions using homemade MATLAB scripts. The LED light source measured from the microscope was used for color recovery. The detailed scripts and methods can be found online: https://ww2.mathworks.cn/matlabcentral/fileexchange/98289-convert-spectrum-to-color.

Below we show the experimental results of the appearance change for different cavity configurations at different intermediate states.

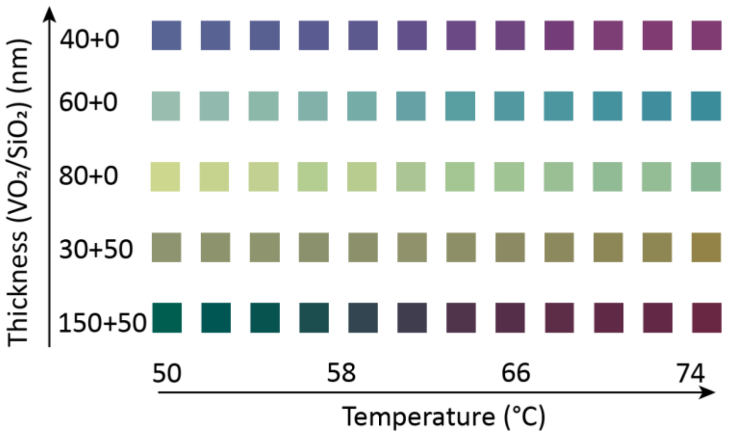

Figure S5. The color appearance for cavities with different cavity configurations.

5. The repeatability and the generation of intermediate states

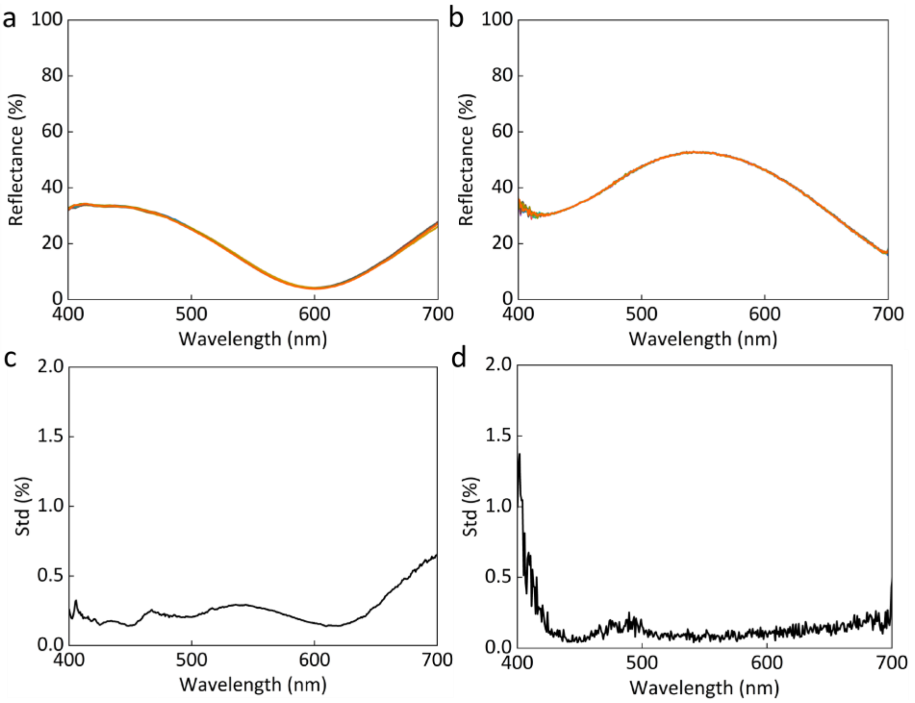

Figure S6. The repeatability of our device. (a)-(b) The reflectance was repeatedly measured 10 times triggering by a hotplate (RT to 80 °C cycles) and voltage source (0 V-2 V cycles). (c)-(d) The standard deviation is less than 1% with a hotplate and 1.5% with voltage stimulus.

6. The intensity modulation of the lossy cavity

The intensity modulation of the lossy cavity is calculated as:

$$M = \frac{|R_m - R_i|}{R_i} \times 100\%$$

where R_m and R_i are the reflectance at metallic and insulating states.

Our devices can show large modulation (even over 100%) within visible regions.

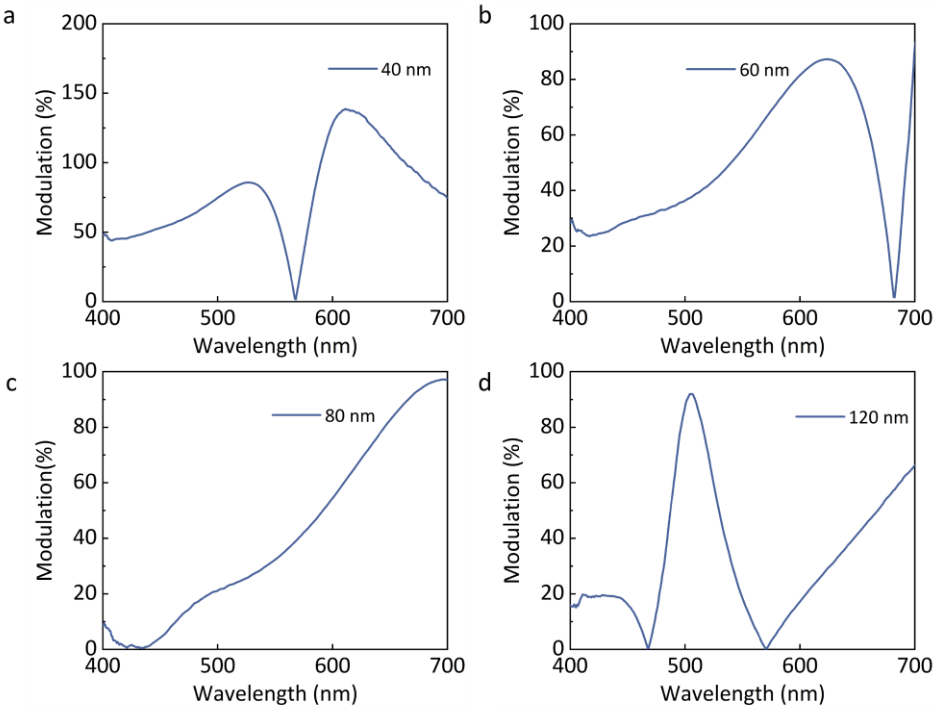

Figure S7. The intensity modulation for our devices with various thicknesses of VO_2.

7. The heat dissipation channels for our devices

The convection and radiation output power for the device is calculated as:

$$P_{conv} = l_1 l_2 h \Delta T = 5.58 \times 10^{-6}(\mathrm{W})$$
$$P_{rad} = l_1 l_2 \sigma T^4 = 1.14 \times 10^{-5}(\mathrm{W})$$

In the above formula, l_1 and l_2 are the length and width of the device indicated in Figure 1, which are 120 μm and 155 μm respectively, h is the heat transfer coefficient with a value of 5 W/(m ^{2}·K). ΔT is the temperature difference between the complete phase transition temperature and room temperature, where ΔT =353-293=60 K is taken. σ is the Stefan Boltzmann constant, with a value of 5.67×10^{-8} W·m^{-2}·K$^{-4,}$ and the value of T is taken as 323 K. As a comparison, the typical input power is $P_{in} = I^2 R = 0.5625(\mathrm{W})$ which is much larger than convection and radiation dissipation power. For this reason, we ignore the convection and radiation dissipation in the calculation.

8. The heat transfer model and calculation

According to the conservation of energy, the heating and cooling process can be estimated by two simple linear models below:

$$I^2 \cdot R \cdot \Delta t_r = c \cdot m \cdot (T_{th} - T_0) + VH + P_{rout} \cdot \Delta t_r \quad (1)$$
$$c \cdot m \cdot (T_0 - T_{th}) + P_{fout} \cdot \Delta t_f = 0 \quad (2)$$

There are three main thermal dissipation channels for the device: conduction, convection and radiation. Due to the small area and low temperature, the convection and radiation are much smaller than the conduction. We only consider thermal conduction for heat dissipation. For simplicity, we can assume there is no in-plane thermal conduction and thermal conduction is mainly spread along the vertical direction. Heat conduction follows Fourier's Law as:

$$Q = -k \cdot S \cdot \frac{\mathrm{d}T}{\mathrm{d}h} \approx -k_e \cdot S \cdot \frac{\mathrm{d}T}{\mathrm{d}h_e} \quad (3)$$

where Q is the thermal flux, $\frac{\mathrm{d}T}{\mathrm{d}h}$ is the temperature gradient along the vertical direction. h means the thickness of various layers. S is the cross-section of each layer. k is the thermal conductivity of each layer. Here we use a single layer with effective thermal conductivity k_e and thickness of h_e to estimate the multilayer device. The effective thermal conductivity k_e is calculated as:

$$\frac{h_e}{k_e} = \frac{h_1}{k_1} + \frac{h_2}{k_2} + \cdots \quad (4)$$

With linear approximation, then E.Q.(3) can be deduced as:

$$Q = -\alpha (k_e \cdot \frac{S}{h_e}) \Delta T$$

Then E.Q.(1) and (2) can be deduced as:

$$I^2 \cdot R \cdot \Delta t_r = c \cdot m \cdot (T_{th} - T_0) + VH + \alpha_1 \cdot (k_e \cdot \frac{S}{h_e}) \Delta T \cdot \Delta t_r$$

$$c \cdot m \cdot (T_0 - T_{th}) + \alpha_2 \cdot (k_e \cdot \frac{S}{h_e}) \Delta T \cdot \Delta t_f = 0$$

where α is a coefficient different for heating and cooling processes, and we estimate it by simulation with COMSOL and assign $\alpha_1 = 0.7$ for the heating process and $\alpha_2 = 0.4$ for the cooling process at t_{SiO2}=2 μm. The latent heat of the phase transition of VO_2 is adopted as 250 J/cm^3 according to reference

22.

Below are the material properties we used in our calculations. We used a sourcemeter (Keithley 2450) to measure the resistance of a single heater and obtained a value of 400 Ohms. Thus, the resistance of 400 Ohms was used to calculate the ITO resistivity, which is used in all simulations.

Table S3. The material properties used in the calculations

	**Thermal capacity** *c*(J/kg·K)	**Density** ρ(g/cm^3)	**Length1** l_1 (μm)	**Length2** l_2 (μm)	**Thickness** *h* (nm)	**Threshold Temperature** T_{th}(°C)	**Initial Temperature** T_0(°C)
**Ag**	235	10.500	120	155	100	/	/
**ITO**	1290	7.090	130	165	50	/	/
**VO_2 (RT)**	3637 [23]	4.339	120	155	40	80	20
**VO_2 (350 K)**	3812 [23]	/	120	155	40	80	20
**SiO_2**	709	2.203	120	155	10	/	/
**Cr**	448	7.150	120	155	10	/	/
**Au**	129	19.3	#	#	#	/	/

# : In different simulations, the geometric dimensions of gold vary. For ultra-fast simulations, the electrode size of gold is 150 × 150 μm², and the electrode size in the array simulation is the same as the actual preparation.

9. Simulation of the dynamic response

The simulation was conducted with COMSOL Multiphysics software. In the simulation, we used thermal insulation boundaries as sidewalls and used heat flux boundaries at the top and initial temperature values at the bottom. All material properties in the simulation were adopted from the built-in library.

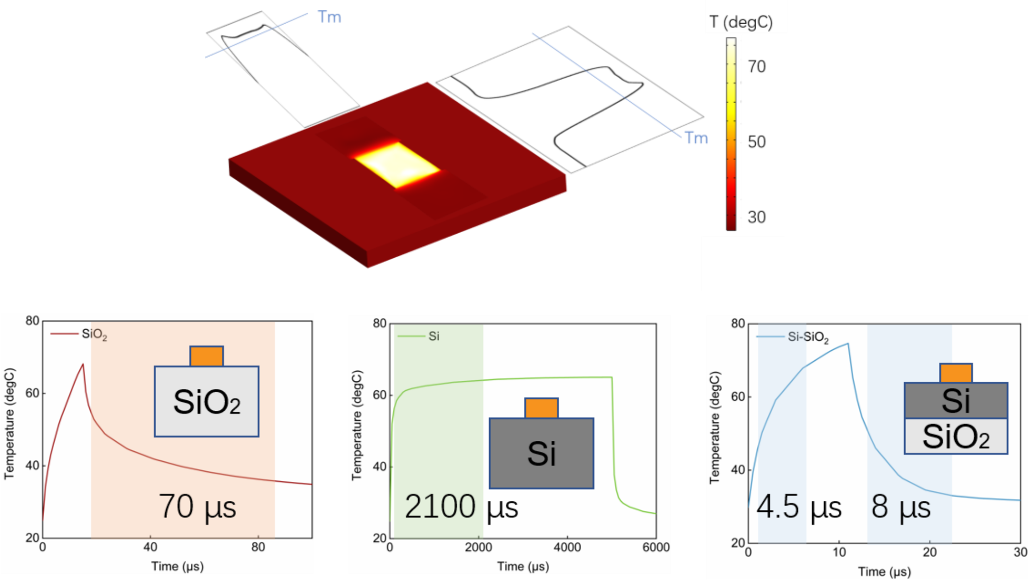

Figure S8. The simulation schematic and results for different substrate configurations.

We simulated the temperature transient response when the substrate was 1 μm silicon dioxide on silicon and the pixel sizes were 5 μm², 10 μm², 25 μm², 35 μm² and 50 μm² (Figure S9(a)). The

corresponding switching times are 236 ns, 250 ns, 360 ns, 650 ns and 11000 ns. In the simulation, the voltage we set is the maximum power under the combination of our signal generator and power amplifier. In actual experiments, excessive power may cause damage to the sample.

The heat capacity of VO_2 will change with the states (see Table S3). However, due to the thin thickness and smaller volume of the VO_2 layer, this heat capacity change will not have a significant impact on the performance of the device. To confirm this, we simulated the switching performance of devices with thermal capacities at both room temperature and 350 K. As shown in Figure S9(b), it has a negligible influence on the performance of the heating and cooling processes.

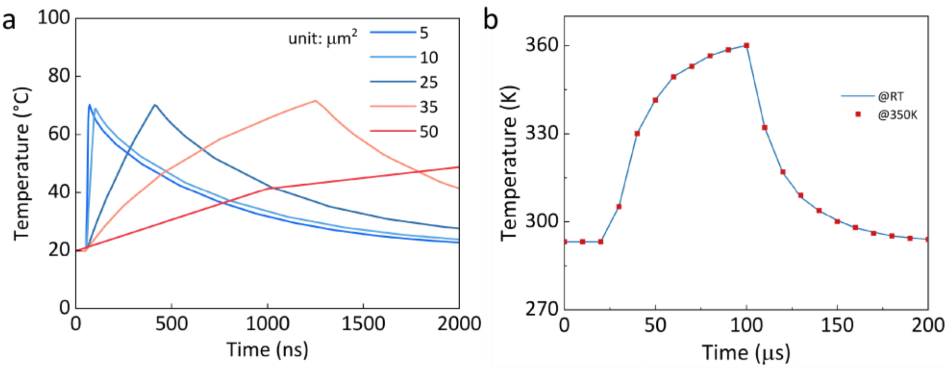

Figure S9. (a) The response time as a function of pixel size on 1 μm silicon dioxide on silicon substrate. (b) The heating and cooling processes with the VO_2 thermal capacities at both room temperature and 350 K.

10. Layout design of the 12×12 matrix and pixel control

The 12×12 matrix device preparation process was divided into five steps, where Figure S10(a) shows the layout of the ITO part for the micro heater and the (SiO_2-Cr-Ag-VO_2) structural color main part above, distinguished by red and purple color, respectively. Figure S10(b) shows the layout of the double-layer electrode. The black area represents the bottom electrode (vertical electrode), followed by the green area of the silica insulation layer at the intersection of the two layers of electrodes, and finally, the blue part represents the top electrode (horizontal electrode). Figure S10(c) magnifies a single unit to better demonstrate the double-layer electrode structure. Figure S10(d) shows the layout of the entire matrix device.

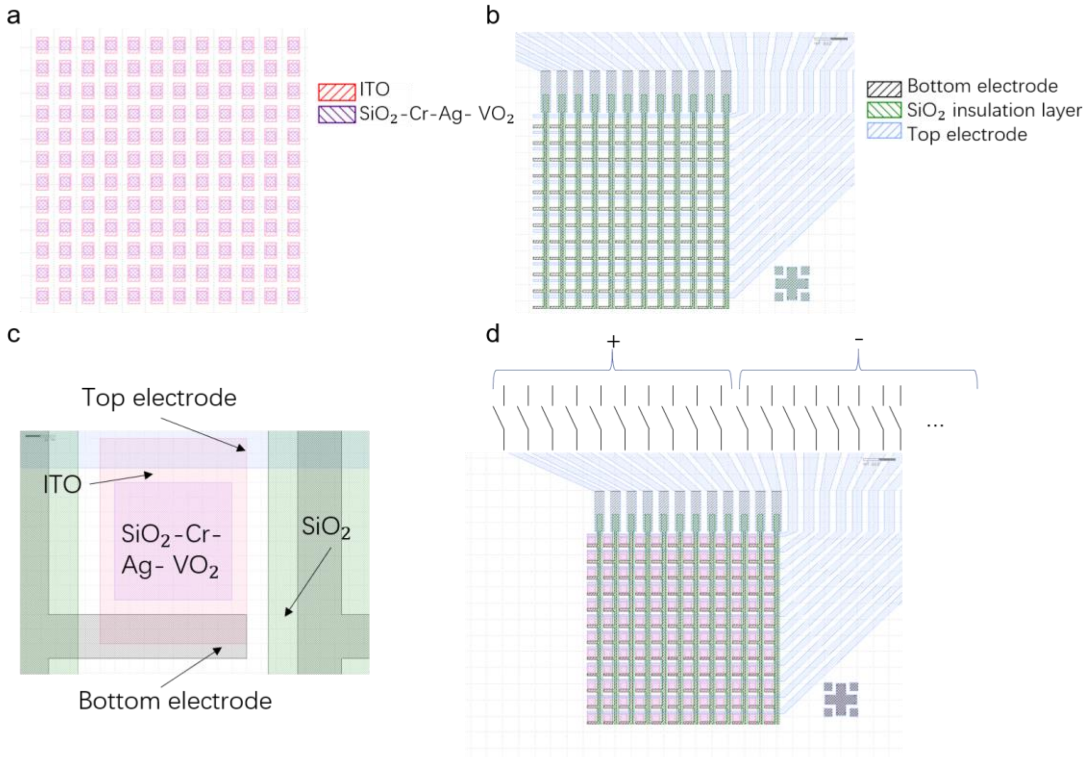

Figure S10. The layout of the pixel matrix.

11. Implementation of matrix control

In subsequent pixel control, all vertical electrodes were connected with a voltage supplier and horizontal electrodes were connected to the ground. Each electrode was independently controlled by a FPGA to generate different signal inputs. We used the IO port of a FPGA (Alinx, AX7020) to generate the LVCMOS33 standard voltage to control the switches (Macrowis) on/off, where 12 IO ports control the on/off of the power supply high-level and the other 12 ports control the on/off of the ground. The maximum switching frequency of the switch was 1 KHz. In the point-by-point scanning, we used a switching rate of 50 ms, while in the line-by-line scanning, we used a switching rate of 100 ms.

12. The uniformity of the matrix

We measured the reflection spectra of 16 pixels at different positions of the matrix (from top-left corner to bottom-right corner) to explore the uniformity of our array, simultaneously in both metallic and insulating states. As shown below, the spectral difference is small, with a standard deviation of less than 1.5% for both the insulating state and the metallic states. These differences may be due to errors in measurement and manufacturing processes.

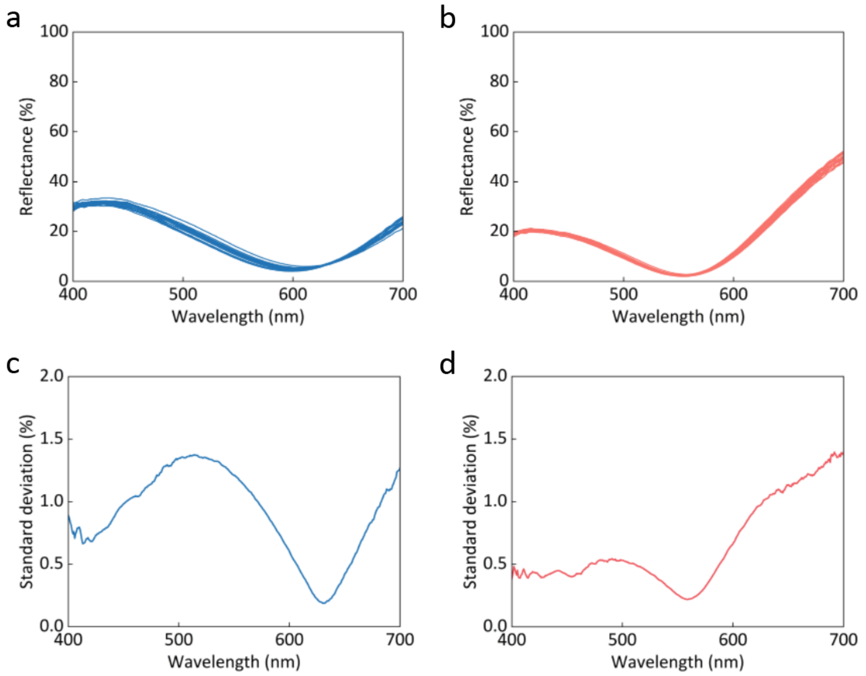

Figure S11. The measured reflective spectra of 16 pixels at different positions for (a) the insulating state and (b) the metallic state. (c)-(d) The standard deviation for insulating and metallic states. The standard deviation is less than 1.5% for both states.

13. The principle of color and spectrum sensing by the active spectral filters

13.1 Color sensing

For color sensing with the tetrachromatic filters, the four reflective filters can be seen as Bayer filters in color cameras. By pre-calibration of the spectra and chromaticity values of these filters on a static state, the chromaticity values of the input signal light can be reconstructed based on the equation below:

$$\begin{bmatrix} \mathcal{R}_1 & \mathcal{G}_1 & \mathcal{B}_1 \\ \mathcal{R}_2 & \mathcal{G}_2 & \mathcal{B}_2 \\ \mathcal{R}_3 & \mathcal{G}_3 & \mathcal{B}_3 \\ \mathcal{R}_4 & \mathcal{G}_4 & \mathcal{B}_4 \end{bmatrix} \times \begin{bmatrix} r & 0 & 0 \\ 0 & g & 0 \\ 0 & 0 & b \end{bmatrix} = \begin{bmatrix} R_1 & G_1 & B_1 \\ R_2 & G_2 & B_2 \\ R_3 & G_3 & B_3 \\ R_4 & G_4 & B_4 \end{bmatrix}$$

where $\mathcal{R}_n$, $\mathcal{G}_n$ and $\mathcal{B}_n$ are chromaticity values of the nth filter pre-measured under a D65 standard light source. r, g and b are chromaticity values of the original signal light. R_n, G_n and B_n are the measured intensity values of the nth filter. r, g and b can be obtained by solving the equation above.

The following figure shows the simulated results for color sensing with the tetrachromatic filters under 450 nm, 500 nm, 550 nm, 600 nm and 650 nm narrowband input light sources (FWHM~47 nm) respectively.

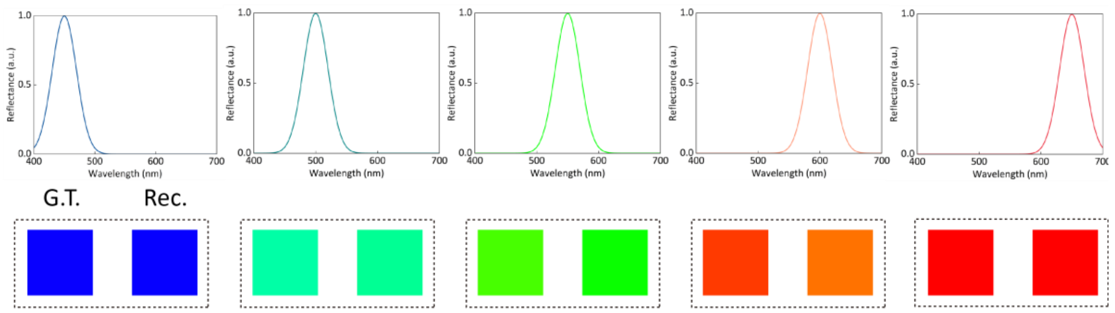

Figure S12. The simulated results for color sensing by the tetrachromatic active spectral filters. G.T. means the ground truth and Rec. represents the reconstructed result.

13.2 Spectrum sensing

As each of the tetrachromatic filters has multiple intermediate states for spectrum modulation, the four filters as a whole can work as a spatiotemporal tunable filter. This filter can used for spectrum detection. For the spectrum detection, the reconstruction principle is represented below:

$$P_{mn} = \int_{\lambda_1}^{\lambda_2} F_{mn}(\lambda)\, I(\lambda)\, \mathrm{d}\lambda$$

where P_{mn} is the detected power of the detector after the mth color filter when the VO_2 filter is at the nth intermediate state, $F_{mn}(\lambda)$ is the response function of the system, λ_1 and λ_2 are the lower and upper limits of the working range. $I(\lambda)$ is the spectrum of incident light.

When working in tuning mode, the above equation can be discretized as:

$$\boldsymbol{P}_{\boldsymbol{mn}} \approx \sum_{q=1}^{Q} \boldsymbol{F}_{\boldsymbol{mn}}(\lambda_q)\, \boldsymbol{I}(\lambda_q), \qquad m = 1 \dots 4, n = 1 \dots N, q = 1 \dots Q$$

where $\boldsymbol{F}_{\boldsymbol{mn}}(\lambda_q) = \boldsymbol{R}_{\boldsymbol{mn}}(\lambda_q)\boldsymbol{D}(\lambda_q)\boldsymbol{L}(\lambda_q)$, $\boldsymbol{R}_{\boldsymbol{mn}}(\lambda_q)$ is the reflective spectrum of the mth color filter at the nth filtering state at λ_q. $\boldsymbol{D}(\lambda_q)$ is the response of the detector, and $\boldsymbol{L}(\lambda_n)$ is the optical response of the system for λ_q. M defines the total number of color filters in a unit, and N is the total intermediate state number for a single VO_2 filter. Q is the discrete wavelength channel that needs to be resolved. Solving these equations by reconstructed algorithms[24] can recover the input signals accurately.

When working in snapshot mode, $\boldsymbol{R}_{\boldsymbol{mn}}(\lambda_q)$ is replaced by the reflective spectrum of the mth color filter for the nth unit pixel at λ_q and N is the total unit pixel number used for spectrum reconstruction.

14. The spectrum detection and reconstruction process

14.1. Setup for spectrum detection

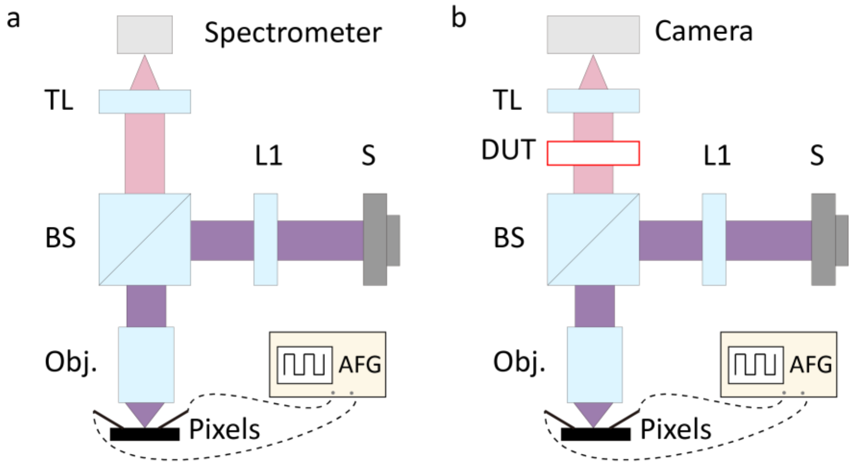

Figure S13. The setup for spectrum detection based on our active pixels. (a) The calibration setup with a spectrometer. (b) The measurement setup with a camera. TL: tube lens; BS: beam splitter; Obj.: objective; L1: lens1; S: source; DUT: device under test. In our paper, the DUT is a series of filters.

14.2. Calibration of the filter arrays

To obtain the spectra of four filters in all different states, calibration work was carried out on an Olympus microscope (BX53M) equipped with a spectrometer (Ocean Insight, QE Pro). The DC electrical signal generated by a signal generator (SIGLENT, SDG1062X) is used to modulate the samples that have been wire bonded, and a spectrometer was used to measure the reflection spectral response at each different voltage state. The synchronization of the spectrometer and signal generator was controlled by a self-built LabVIEW script.

14.3. Measurement

Measurements for commercial filters were done on the same microscope system. By inserting various commercial filters into the incident light path, the reflected images were captured by the black and white camera (ZWO, ASI432MM). For different filters, we needed to adjust the exposure time of the camera so that the intensity of all pixels was below the saturated value.

14.4. Reconstruction

The recovery of the measured spectra was done by MATLAB R2023a software. Because each color filter occupied ~55×55 pixels on the camera. Considering the measurement error, only the central 30×30 pixels for a filter were averaged for reconstruction. This averaged result was used as the detected power matrix $\boldsymbol{P}_{\boldsymbol{mn}}(\lambda_q)$. The response matrix $\boldsymbol{R}_{\boldsymbol{mn}}(\lambda_q)$ with size 144×101 was obtained in the calibration session, that the rows represent different filters (M = 4 and N = 36) and the columns represent wavelengths channel (Q = 101). The optical response $\boldsymbol{L}(\lambda_q)$ and the quantum efficiency $\boldsymbol{D}(\lambda_q)$ of the camera were fixed and included in the spectrum of incident light $\boldsymbol{I}(\lambda_q)$ in the process of reconstruction.

To prevent over-fitting, we used the ridge regression and LASSO regression models in the solving process. The following formula is the cost function J:

$$J(I) = (RI - P)^2 + \lambda_1 \Sigma |I|^2 + \lambda_2 \Sigma |I|$$

The first term of J is the linear regression model, the second term is the L_1 regularization, the third term is the L_2 regularization, and λ_1 and λ_2 are the regularization coefficients of the two, respectively. The CVX tool was used to solve the equation above.[24]

The root mean square error (RMSE) between the reference and reconstructed spectrum is calculated as:

$$RMSE = \sqrt{\frac{1}{Q}\sum_{q=1}^{Q}\left(\boldsymbol{I}(\lambda_q) - \boldsymbol{I}'(\lambda_q)\right)^2}$$

The fidelity (S) is calculated as:

$$S = \frac{\boldsymbol{I}\cdot\boldsymbol{I}'}{||\boldsymbol{I}||\,||\boldsymbol{I}'||} \times 100\% = \frac{\sum_{q=1}^{Q}\boldsymbol{I}(\lambda_q)\times\boldsymbol{I}'(\lambda_q)}{\sqrt{\sum_{q=1}^{Q}\boldsymbol{I}(\lambda_q)^2}\times\sqrt{\sum_{q=1}^{Q}\boldsymbol{I}'(\lambda_q)^2}} \times 100\%$$

where I' is the reference spectrum and I is the recovered spectrum. Q denotes the number of wavelength sampling points.

15. The sneak current in the cross-bar scheme

The sneak current can have a significant influence on the performance of the device. To analyze this effect, a simple circuit model was used for simulation.[25] In this model, the whole device can be simplified as the figure shown below.

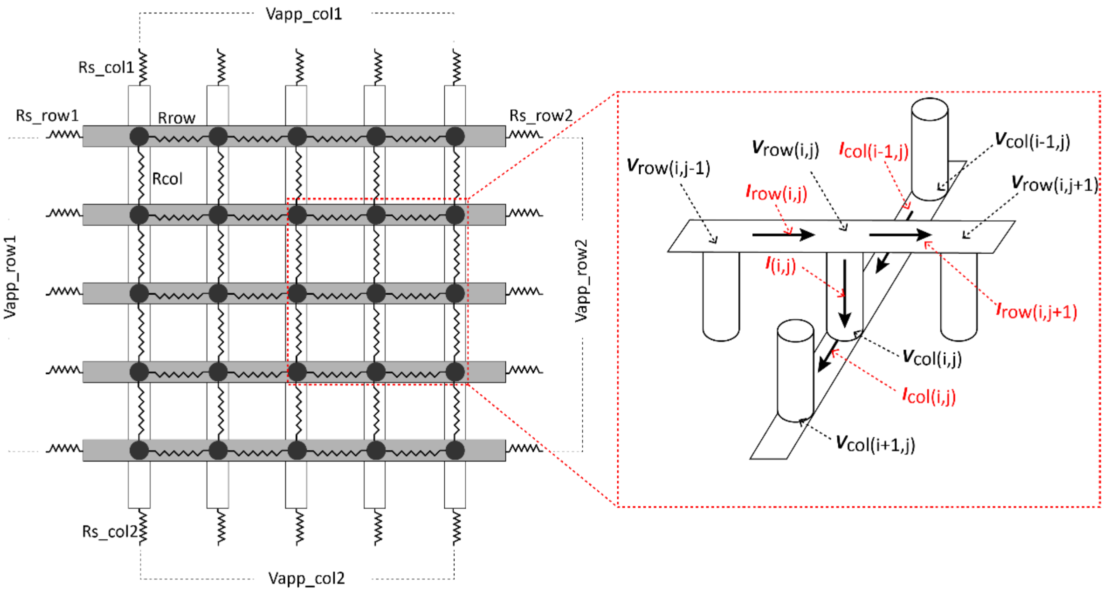

Figure S14. The proposed circuit model based on Kirchhoff's law.

Here, R is the resistance of the single heater, Rs_row1/Rs_row2 are the resistances between the applied source $Vapp\_row1/\ Vapp\_row2$ and the row lines of the device, $Rs\_col1/\ Rs\_col2$ are the resistances between the applied source $Vapp\_col1/\ Vapp\_col2$ and the column lines of the device. The R_row and R_col are the resistances between adjacent heaters for row lines and column lines. The $V\mathrm{row}(i,j)$ is the row line voltage applied onto the heater at i row and j column. $V\mathrm{col}(i,j)$ is the column line voltage applied onto the heater at i row and j column. $I(i,j)$ is the current passing through the heater at i row and j column. $I\mathrm{col}(i,j)$ is the current passing through the column lines between heater $R(i,j)$ and heater $R(i-1,j)$. $I\mathrm{row}(i,j)$ is the current passing through the row lines between heater $R(i,j)$ and heater $R(i,j-1)$.

According to Kirchhoff's law, the current through each node (heater) can be written as:

$$\frac{V_{\mathrm{row}}(i,j) - V_{\mathrm{row}}(i,j+1)}{R_{\mathrm{row}}} + \frac{V_{\mathrm{row}}(i,j) - V_{\mathrm{col}}(i,j)}{R(i,j)} = \frac{V_{\mathrm{row}}(i,j-1) - V_{\mathrm{row}}(i,j)}{R_{\mathrm{row}}}, \quad (1 \le i \le m, \; 2 \le j \le n-1);$$

$$\frac{V_{\mathrm{row}}(i,j) - V_{\mathrm{row}}(i,j+1)}{R_{\mathrm{row}}} + \frac{V_{\mathrm{row}}(i,j) - V_{\mathrm{col}}(i,j)}{R(i,j)} = \frac{V_{\mathrm{APP\_row1}}(i) - V_{\mathrm{row}}(i,j)}{R_{\mathrm{s\_row1}}(i)}, \quad (1 \le i \le m, \; j = 1);$$

$$\frac{V_{\mathrm{row}}(i,j) - V_{\mathrm{APP\_row2}}(i)}{R_{\mathrm{s\_row2}}(i)} + \frac{V_{\mathrm{row}}(i,j) - V_{\mathrm{col}}(i,j)}{R(i,j)} = \frac{V_{\mathrm{row}}(i,j-1) - V_{\mathrm{row}}(i,j)}{R_{\mathrm{row}}}, \quad (1 \le i \le m, \; j = n);$$

$$\frac{V_{\mathrm{col}}(i-1,j) - V_{\mathrm{col}}(i,j)}{R_{\mathrm{col}}} + \frac{V_{\mathrm{row}}(i,j) - V_{\mathrm{col}}(i,j)}{R(i,j)} = \frac{V_{\mathrm{col}}(i,j) - V_{\mathrm{col}}(i+1,j)}{R_{\mathrm{col}}}, \quad (2 \le i \le m-1, \; 1 \le j \le n);$$

$$\frac{V_{\mathrm{APP\_col1}}(j) - V_{\mathrm{col}}(i,j)}{R_{\mathrm{s\_col1}}(j)} + \frac{V_{\mathrm{row}}(i,j) - V_{\mathrm{col}}(i,j)}{R(i,j)} = \frac{V_{\mathrm{col}}(i,j) - V_{\mathrm{col}}(i+1,j)}{R_{\mathrm{col}}}, \quad (i = 1, \; 1 \le j \le n);$$

$$\frac{V_{\mathrm{col}}(i-1,j) - V_{\mathrm{col}}(i,j)}{R_{\mathrm{col}}} + \frac{V_{\mathrm{col}}(i,j) - V_{\mathrm{col}}(i,j)}{R(i,j)} = \frac{V_{\mathrm{col}}(i,j) - V_{\mathrm{APP\_col2}}(i)}{R_{\mathrm{s\_col2}}(j)}, \quad (i = m, \; 1 \le j \le n);$$

The equations above can be rewritten in matrix format as:

$$\begin{bmatrix} \boldsymbol{A} & \boldsymbol{B} \\ \boldsymbol{C} & \boldsymbol{D} \end{bmatrix} \boldsymbol{V} = \boldsymbol{E}$$

where $\boldsymbol{A}$, $\boldsymbol{B}$, $\boldsymbol{C}$, $\boldsymbol{D}$ are all $mn \times mn$ matrices, $\boldsymbol{V}$ is a $2mn \times 1$ vector and $\boldsymbol{E}$ is a $2mn \times 1$ vector.

Matrices $\boldsymbol{A}$, $\boldsymbol{B}$, $\boldsymbol{C}$, and $\boldsymbol{D}$ can be further written as:

$$A = \begin{bmatrix} \boldsymbol{A_1} & 0 & \cdots & 0 \\ 0 & \boldsymbol{A_2} & \cdots & 0 \\ \vdots & \vdots & \ddots & \vdots \\ 0 & 0 & \cdots & \boldsymbol{A_m} \end{bmatrix}$$

$\boldsymbol{A_i}(1 \le i \le m)$ are $n \times n$ matrices as shown below:

$$\boldsymbol{A_i} = \begin{bmatrix} \frac{1}{R_{\mathrm{s\_row1}}(i)} + \frac{1}{R(i,1)} + \frac{1}{R_{\mathrm{row}}} & \frac{-1}{R_{\mathrm{row}}} & 0 & \cdots & 0 \\ \frac{-1}{R_{\mathrm{row}}} & \frac{1}{R(i,2)} + \frac{2}{R_{\mathrm{row}}} & \frac{-1}{R_{\mathrm{row}}} & \cdots & 0 \\ \vdots & \vdots & \vdots & \ddots & \frac{-1}{R_{\mathrm{row}}} \\ 0 & \cdots & 0 & \frac{-1}{R_{\mathrm{row}}} & \frac{1}{R_{\mathrm{s\_row2}}(i)} + \frac{1}{R(i,n)} + \frac{1}{R_{\mathrm{row}}} \end{bmatrix}$$

$$\boldsymbol{B} = \begin{bmatrix} \boldsymbol{B_1} & 0 & \cdots & 0 \\ 0 & \boldsymbol{B_2} & \cdots & 0 \\ \vdots & \vdots & \ddots & \vdots \\ 0 & 0 & \cdots & \boldsymbol{B_m} \end{bmatrix}$$

$\boldsymbol{B_i}(1 \le i \le m)$ are $n \times n$ matrices as shown below:

$$\boldsymbol{B_i} = \begin{bmatrix} \frac{-1}{R(i,1)} & 0 & \cdots & 0 \\ 0 & \frac{-1}{R(i,2)} & \cdots & 0 \\ \vdots & \vdots & \ddots & \vdots \\ 0 & 0 & \cdots & \frac{-1}{R(i,n)} \end{bmatrix}$$

$$\boldsymbol{C} = \begin{bmatrix} \boldsymbol{C_1} \\ \boldsymbol{C_2} \\ \vdots \\ \boldsymbol{C_n} \end{bmatrix}$$

$\boldsymbol{C_j}(1 \leq j \leq n)$ are $m \times mn$ matrices as shown below:

$$\boldsymbol{C_j} = \begin{cases} C_j(i, n(i-1)+j) = \frac{1}{R(i,j)} \quad 1 \leq i \leq m \\ \text{The rest elements are "0"} \end{cases}$$

$$\boldsymbol{D} = \begin{bmatrix} \boldsymbol{D_1} \\ \boldsymbol{D_2} \\ \vdots \\ \boldsymbol{D_n} \end{bmatrix}$$

$\boldsymbol{D_j}(1 \leq j \leq n)$ are $m \times mn$ matrices as shown below:

$$\boldsymbol{D_j} = \begin{cases} D_j(i,j) = \left(\frac{-1}{R_{\text{s\_col1}}(j)} + \frac{-1}{R_{\text{col}}} + \frac{-1}{R(i,j)}\right), D_j(i, ni+j) = \frac{1}{R_{\text{col}}} \ (i = 1; 1 \leq j \leq n) \\ D_j(i, n(i-2)+j) = \frac{1}{R_{\text{col}}}, D_j(i, n(i-1)+j) = \left(\frac{-1}{R_{\text{col}}} + \frac{-1}{R(i,j)} + \frac{-1}{R_{\text{col}}}\right), D_j(i, ni+j) = \frac{1}{R_{\text{col}}} \ (2 \leq i \leq m-1; 1 \leq j \leq n) \\ D_j(i, n(i-2)+j) = \frac{1}{R_{\text{col}}}, \ D_j(i, n(i-1)+j) = \left(\frac{-1}{R_{\text{s\_col2}}(j)} + \frac{-1}{R(i,j)} + \frac{-1}{R_{\text{col}}}\right) \ (i = m; 1 \leq j \leq n) \\ \text{The rest elements are "0"} \end{cases}$$

$$\boldsymbol{V} = \begin{bmatrix} V_{\text{row}}(1,1) \ldots V_{\text{row}}(1,n), \ldots, V_{\text{row}}(m,1) \ldots V_{\text{row}}(m,n), \\ V_{\text{col}}(1,1) \ldots V_{\text{col}}(1,n), \ldots, V_{\text{col}}(m,1) \ldots V_{\text{col}}(m,n) \end{bmatrix}^T$$

$$\boldsymbol{E} = \begin{bmatrix} \boldsymbol{E_W} \\ \boldsymbol{W_B} \end{bmatrix}$$

$$\boldsymbol{E_W} = \begin{bmatrix} \boldsymbol{E_{W1}} \\ \boldsymbol{E_{W2}} \\ \vdots \\ \boldsymbol{E_{Wm}} \end{bmatrix}$$

$$\boldsymbol{E_B} = \begin{bmatrix} \boldsymbol{E_{B1}} \\ \boldsymbol{E_{B2}} \\ \vdots \\ \boldsymbol{E_{Bn}} \end{bmatrix}$$

$$\boldsymbol{E_{wi}} = \begin{bmatrix} \frac{V_{\text{APP\_row1}}(1,i)}{R_{\text{s\_row1}}(2,i)} \\ 0 \\ 0 \\ \vdots \\ \frac{V_{\text{APP\_row2}}(n-1,i)}{R_{\text{s\_row2}}(n,i)} \end{bmatrix}, (1 \leq i \leq m)$$

$$
\boldsymbol{E_{Bj}} = \begin{bmatrix} \dfrac{V_{\text{APP\_col1}}(1,j)}{R_{\text{s\_col1}}(2,j)} \\ 0 \\ 0 \\ \vdots \\ \dfrac{V_{\text{APP\_col2}}(m-1,j)}{R_{\text{s\_col2}}(m,j)} \end{bmatrix}, (1 \le j \le n)
$$

In the simulation, the size of the matrix was set as 12×12. The resistance for line-wire and column wire are 2 Ω and 8.5 Ω respectively. The applied voltage was the same with an experiment result of 1.8 V. The resistance for each heater was set as 400 Ω. In Figure S15, we compare the calculated effective resistance distribution (Figure S15(b)) with the measured ones in the array (Figure S15(a)). As can be seen, the calculated results based on the model fit quite well with the measured resistances, both are smaller than a single heater (400 Ω). This is an evident result of the sneak current path. In addition, the resistance in both simulation and experiment varies for each heater at different positions. We ascribe it to the line-wire resistance between each heater.

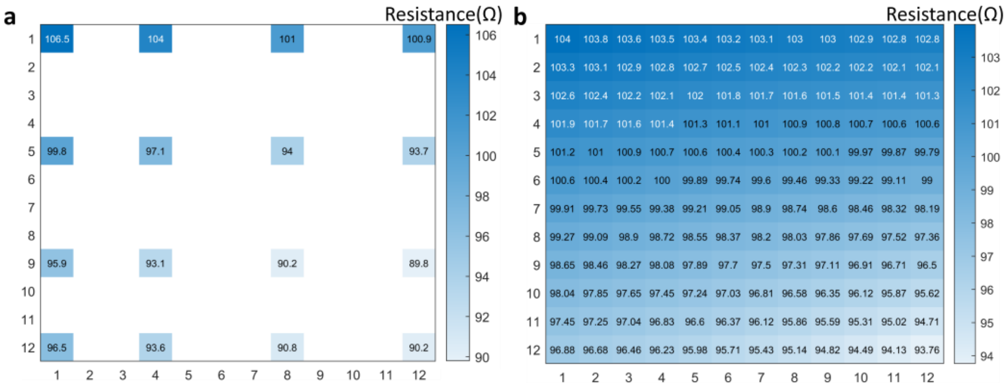

Figure S15. (a) The measured resistance distribution in the array and (b) the simulated results based on the proposed circuit model.

Using this model, we check the sneak currents at different positions (Figure S16). It shows the current passing through the '*set*' heater can be at least two times larger than the largest sneak current. This result ensures that the VO_2 cavities can be addressed individually, with little crosstalk caused by the sneak current. Due to the sneak current, the effective power consumption is larger than expected. The measured power consumption is $P = UI = 1.8\text{ V} \times 15\text{ mA} = 27\text{ mW}$ for a heater in the array, close to the simulated results of ~30 mW for the pixelated device (see figure below). As a comparison, for a single heater, the calculated power consumption is $P = UI = 1.8\text{ V} \times 2.2\text{ mA} = 3.96\text{ mW}$. This confirms that the sneak current would lead to waste power dissipation.

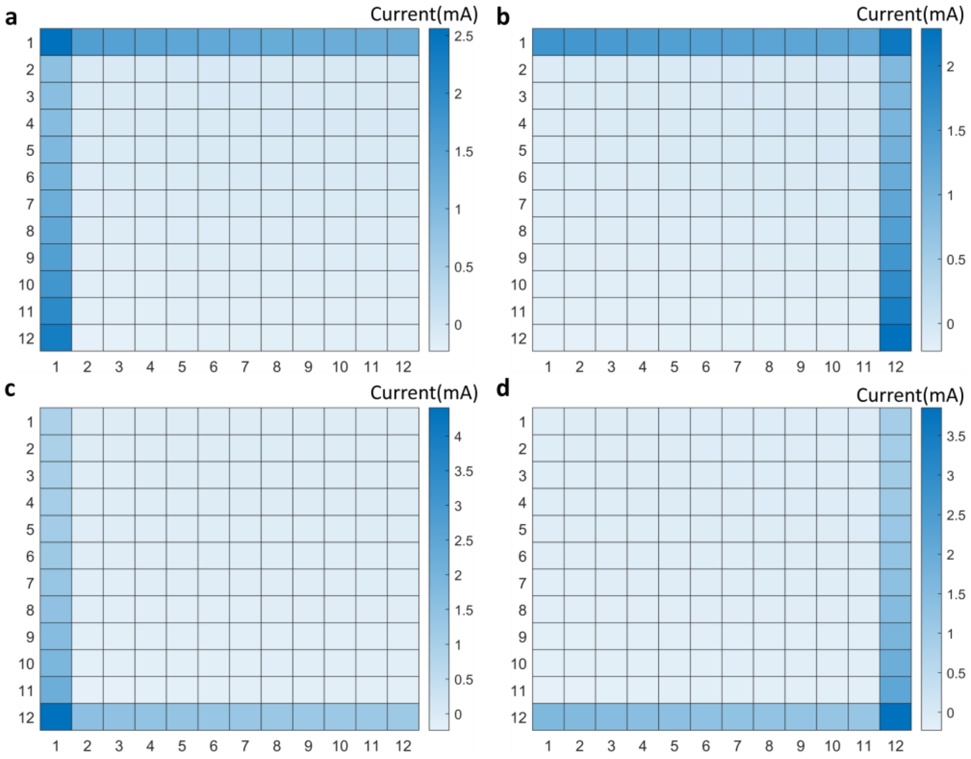

Figure S16. a-d. The current passing through each heater in the array considering the sneak current and resistance of the connecting lines when applying voltage on the heaters at positions: (1,1), (1,12), (12,1), (12,12). The total currents are: 17.5, 17.3, 19.2 and 18.6 mA, and the total power consumptions are: 31.5, 31.2, 34.6 and 33.4 mW. The *x*-axis label means the row numbers and the *y*-axis label means the column numbers.

Moreover, to explore the behavior of the heater in an array (e.g. in the structural color display), we simulated the electrical and thermal characteristics of a single pixel in the array, accounting for the sneak current with COMSOL Multiphysics. In the simulation, we used ITO as the heater. Its conductivity was measured using the four-probe method. A value of 5×10^4 S/m was used for it in the simulation. In the array simulation, the material of the wire was gold, and the material parameters were chosen from the built-in material library. The thickness of the bottom gold wire was 100 nm, and the thickness of the top gold wire was 350 nm, a width of 15 μm, which were consistent with the parameters in our experiment. We added a positive voltage on a terminal and the ground voltage on another terminal of the selected row. At the same time, in order to simulate the situation of an open circuit in the other ports, we set a boundary layer with a conductivity of 1×10^{-10} S/m. For all external boundaries, we set open thermal boundary conditions except for the quartz glass substrate and top surfaces where a heat flux condition with an ambient temperature of T_0 = 293 K and a convective heat transfer coefficient h of 5 W/(m^2·K) were considered. Our numerical simulation results have shown the effective current (about 20 mA) for a heater in the array. For comparison, we have also simulated the electric current for a single heater of the same geometry and obtained a value of 4.5 mA. The simulated effective current (about 20 mA) for a heater in an array deviates significantly from (much larger than) the electric current (4.5 mA) for a single heater.

In this paper, we use cross-bar architecture as a prototype demonstration. But for a larger scale, transistors could be incorporated into the pixel to provide selectivity and prevent drive signals from

effectively short-circuiting via multiple "sneak paths" through neighboring pixels.

16. The simulated and experimental results for thermal crosstalk

To further investigate the impact of thermal crosstalk on our device, we selected pixels from the first row and first column (Pixel #1) in the upper left corner of the array and the second row and first column (Pixel #2) by applying a series of different voltage signals. As can be seen in Figure S17a, at 1.6 V, Pixel #1 begins to undergo a phase transition, while Pixel #2 remains unchanged. At 1.8 V, Pixel #1 undergoes a complete phase transition, while Pixel #2 remains unchanged. At 2.3V, Pixel #1 undergoes a complete phase transition and Pixel #2 begins to undergo a phase transition. The actual phase transition can be fully observed through microscopic images and the corresponding spectra (Figure S17b), indicating the little crosstalk between adjacent pixels. Furthermore, we also simulated the thermal crosstalk of the array, and the simulated temperature matched well with the actual phase transition situation (based on previous experiments, it was believed that phase transition began around 325 K and was completed around 340 K).

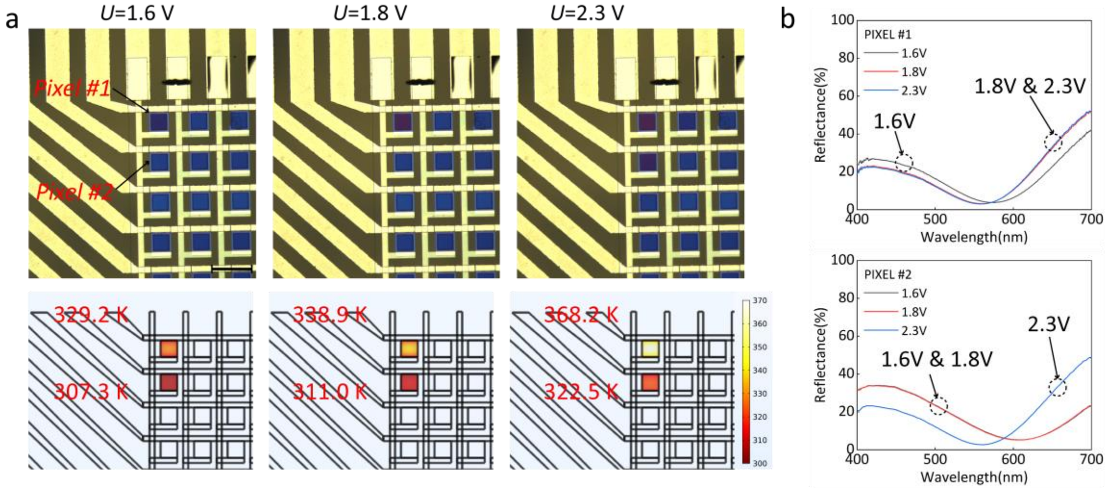

Figure S17. The thermal crosstalk of our matrix. (a) The measured optical images and the corresponding simulated temperature distribution. Scale bar: 100 µm. (b) The corresponding reflectance spectra for Pixel #1 and Pixel #2.

17. The PCM-based structural color filters

Compared with other structural color filters made of PCM, the proposed filter has priorities over modulation frequency, pixel-addressed ability and lifetime over these devices. Due to the simple fabrication process and configuration, it shows low cost and simple system complexity.

Table S4. Comparison of dynamic structural color displays based on PCMs.

**Refs.**	**PCM Type**	**Stimuli**	**Pixel-addressability**	**Reversibility**	**Speed**	**Endurance**	**Year**
[26]	$Ge_2Sb_2Te_5$	Electrical tip	NO	**YES**	N. A.	N. A.	2014
[27]	$Ge_2Sb_2Te_5$	Laser	NO	**YES**	2 Hz	N. A.	2015
[28]	$Ge_2Sb_2Te_5$	Electrical tip	NO	NO	N. A.	N. A.	2016
[29]	$Ge_2Sb_2Te_5$	Electrical tip	NO	**YES**	N. A.	N. A.	2016

[30]	VO_2	Thermal	NO	**YES**	N. A.	N. A.	2018
[22]	VO_2	Laser	NO	**YES**	N. A.	N. A.	2018
[31]	Sb_2S_3	Electrical	NO	NO	N. A.	N. A.	2019
[32]	GeTe	Laser	NO	N. A.	N. A.	N. A.	2019
[33]	Sb_2S_3	Laser	NO	**YES**	N. A.	>10	2020
[34]	VO_2	Thermal&doping	NO	YES	N. A.	N. A.	2020
[35]	VO_2	Thermal	NO	YES	N. A.	N. A.	2021
[13]	Sb_2S_3	Electrical	NO	NO	N. A.	N. A.	2021
[36]	Sb_2S_3	Electrical	NO	NO	N. A.	N. A.	2021
**This work**	**VO_2**	**Electrical**	**YES**	**YES**	**>70 kHz**	**>one million**	**2024**

18. The transmissive spectral filter and filters for the infrared band

As an example, here we show a possible design approach for transmissive filters. In the filter, the VO_2 is laid beneath a pixel-coded nanorod metasurface made of Aluminum (Al). By optimal design, the metasurface combined with VO_2 can have distinct transmissive responses at low (insulating phase, dashed lines) and high temperatures (metallic phase, solid lines). In the design, the width for each nanorod of Al was 100 nm. The thicknesses for Al and VO_2 were 30 nm and 20 nm respectively. A single filter consists of 25 pixels with C4 symmetry (see Figure S18(a)). The whole unit cell consists of four different filters with a total number of 100 pixels on a heater. The simulated transmittance for these filters is shown in Figure S18(b).

The above filter design can also work in the infrared band. Figure S18(c) shows the transmissive spectra of four filters in the NIR band (700-900 nm). We used these filters for spectrum reconstruction simulation. In the simulation, a Gaussian input signal centered at 800 nm with a bandwidth of ~47 nm was used. The simulation results show a good reconstruction accuracy even with a noise ratio of around 5% (Figure S18(d)).

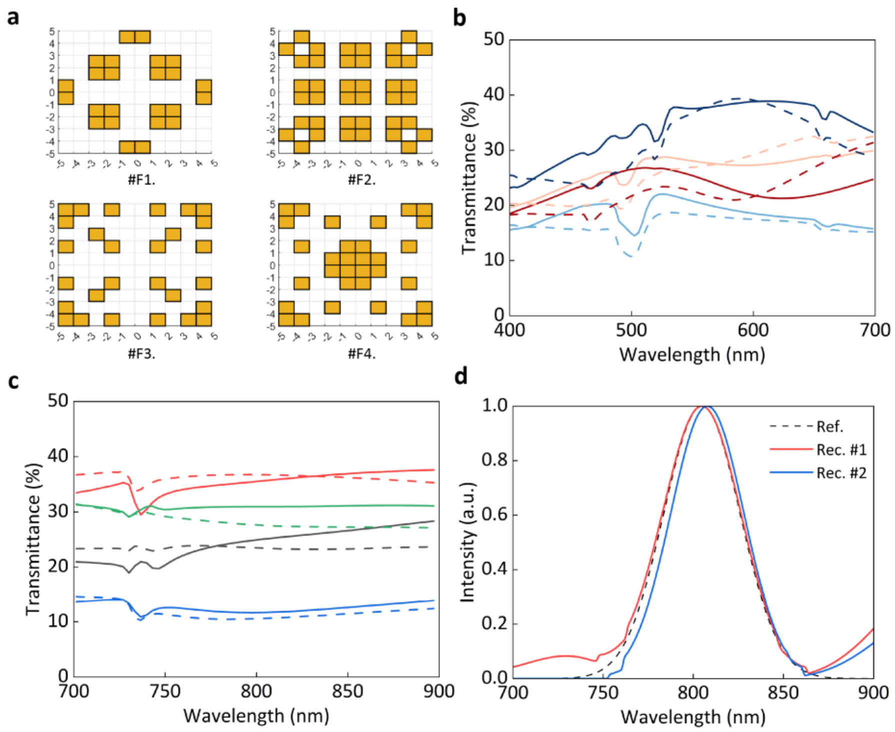

Figure S18. (a) The layout of the four unit pixels for transmissive spectral filters. (b) The corresponding transmissive spectra for the four filters under insulating (solid lines) and metallic (dashed lines) states. (c) The simulated transmissive spectra at the NIR range. (d) The reconstruction results for the four filters in (c). Rec. #1 is the reconstructed result with an intensity noise of 1%. Rec. #2 is the reconstructed result with a noise intensity of 5%. The reconstruction RMSE can be around 0.056 even for the input signal with a noise ratio of 5%.

References

1. Driencourt, L.; Federspiel, F.; Kazazis, D.; Tseng, L.-T.; Frantz, R.; Ekinci, Y.; Ferrini, R.; Gallinet, B., Electrically Tunable Multicolored Filter Using Birefringent Plasmonic Resonators and Liquid Crystals. ACS Photonics 2020.
2. Yao, Y.; Shankar, R.; Kats, M. A.; Song, Y.; Kong, J.; Loncar, M.; Capasso, F., Electrically Tunable Metasurface Perfect Absorbers for Ultrathin Mid-Infrared Optical Modulators. Nano Letters 2014.
3. Wu, P. C.; Pala, R. A.; Kafaie Shirmanesh, G.; Cheng, W.-H.; Sokhoyan, R.; Grajower, M.; Alam, M. Z.; Lee, D.; Atwater, H. A., Dynamic beam steering with all-dielectric electro-optic III–V multiple-quantum-well metasurfaces. Nature communications 2019, 10 (1), 3654.
4. Damgaard-Carstensen, C.; Thomaschewski, M.; Ding, F.; Bozhevolnyi, S. I., Electrical Tuning of Fresnel Lens in Reflection. ACS Photonics 2021.
5. Xiong, K.; Tordera, D.; Emilsson, G.; Olsson, O.; Linderhed, U.; Jonsson, M. P.; Dahlin, A. B., Switchable Plasmonic Metasurfaces with High Chromaticity Containing Only Abundant Metals. Nano Letters 2017.
6. Zangeneh Kamali, Khosro, et al. "Electrically programmable solid-state metasurfaces via flash localised heating." Light: Science & Applications 12.1 (2023): 40.
7. Chen, Benwen, et al. "Programmable terahertz metamaterials with non-volatile memory." Laser & Photonics Reviews 16.4 (2022): 2100472.
8. Wang, Yifei, et al. "Electrical tuning of phase-change antennas and metasurfaces." Nature Nanotechnology 16.6 (2021): 667-672.
9. Zhang, Yifei, et al. "Electrically reconfigurable non-volatile metasurface using low-loss optical phase-change material." Nature Nanotechnology 16.6 (2021): 661-666.
10. Chen, Benwen, et al. "Electrically addressable integrated intelligent terahertz metasurface." Science Advances 8.41 (2022): eadd1296.
11. Fang, Zhuoran, et al. "Non-volatile Phase-only Transmissive Spatial Light Modulators." arXiv preprint arXiv:2307.12103 (2023).
12. Abdollahramezani, Sajjad, et al. "Electrically driven reprogrammable phase-change metasurface reaching 80% efficiency." Nature Communications 13.1 (2022): 1696.
13. Hemmatyar, Omid, et al. "Enhanced meta-displays using advanced phase-change materials." arXiv preprint arXiv:2107.12159 (2021).
14. Youngblood, Nathan, et al. "Reconfigurable low-emissivity optical coating using ultrathin phase change materials." ACS Photonics 9.1 (2021): 90-100.
15. Shu, Fang-Zhou, et al. "Electrically driven tunable broadband polarization states via active metasurfaces based on joule-heat-induced phase transition of vanadium dioxide." Laser & Photonics Reviews 15.10 (2021): 2100155.
16. Fang, Zhuoran, et al. "Non-volatile Phase-only Transmissive Spatial Light Modulators." arXiv preprint arXiv:2307.12103 (2023).
17. Popescu, Cosmin Constantin, et al. "Electrically Reconfigurable Phase-Change Transmissive Metasurface." Advanced Materials (2024): 2400627.
18. Chen, Rui, et al. "Non-volatile electrically programmable integrated photonics with a 5-bit operation." Nature Communications 14.1 (2023): 3465.
19. Wei, Maoliang, et al. "Monolithic back-end-of-line integration of phase change materials into

foundry-manufactured silicon photonics." Nature Communications 15.1 (2024): 2786.
20. Louloudakis, Dimitrios, et al. "Atmospheric pressure chemical vapor deposition of amorphous tungsten doped vanadium dioxide for smart window applications." Advanced Materials Letters 7.3 (2016): 192-196.
21. Outón, J., et al. "Tracking the optical constants of porous vanadium dioxide thin films during metal–insulator transition: influence of processing conditions on their application in smart glasses." Applied Surface Science 580 (2022): 152228.
22. Dong, Kaichen, et al. "A Lithography-Free and Field-Programmable Photonic Metacanvas." Advanced Materials 30.5 (2018): 1703878.
23. Chase Jr, Malcolm W. "JANAF thermochemical tables." J. Phys. Chem. Ref. Data (1985).
24. M. Grant, S. Boyd, CVX: Matlab software for disciplined convex programming, version 2.0 beta, http://cvxr.com/cvx (accessed: September 2013).
25. Chen, An. "A comprehensive crossbar array model with solutions for line resistance and nonlinear device characteristics." IEEE Transactions on Electron Devices 60.4 (2013): 1318-1326.
26. Hosseini, Peiman, C. David Wright, and Harish Bhaskaran. "An optoelectronic framework enabled by low-dimensional phase-change films." Nature 511.7508 (2014): 206-211.
27. Schlich F F, Zalden P, Lindenberg A M, et al. Color switching with enhanced optical contrast in ultrathin phase-change materials and semiconductors induced by femtosecond laser pulses. ACS photonics, 2015, 2(2): 178-182.
28. Yoo S, Gwon T, Eom T, et al. Multicolor changeable optical coating by adopting multiple layers of ultrathin phase change material film. ACS Photonics, 2016, 3(7): 1265-1270.
29. Ríos C, Hosseini P, Taylor R, et al. Color depth modulation and resolution in Phase-Change Material nano-displays. Advanced Materials, 2016, 28(23).
30. Shu F Z, Yu F F, Peng R W, et al. Dynamic plasmonic color generation based on phase transition of vanadium dioxide. Advanced Optical Materials, 2018, 6(7): 1700939.
31. Dong W, Liu H, Behera J K, et al. Wide bandgap phase change material tuned visible photonics. Advanced Functional Materials, 2019, 29(6): 1806181.
32. Carrillo S G C, Trimby L, Au Y Y, et al. A nonvolatile phase-change metamaterial color display. Advanced Optical Materials, 2019, 7(18): 1801782.
33. Hailong Liu et al., Rewritable Laser switching color nanoprints in antimony trisulfide films.Sci. Adv.6, eabb7171(2020).DOI:10.1126/sciadv.abb7171
34. Duan X, White S T, Cui Y, et al. Reconfigurable multistate optical systems enabled by VO2 phase transitions. ACS photonics, 2020, 7(11): 2958-2965.
35. Zhao J, Zhou Y, Huo Y, et al. Flexible dynamic structural color based on an ultrathin asymmetric Fabry-Perot cavity with phase-change material for temperature perception. Optics Express, 2021, 29(15): 23273-23281.
36. Sreekanth K V, Medwal R, Srivastava Y K, et al. Dynamic color generation with electrically tunable thin film optical coatings. Nano letters, 2021, 21(23): 10070-10075.
